# Eco-friendly green synthesis of silver nanoparticles from guajava leaves extract for controlling organophosphorus pesticides hazards, characterization, and in-vivo toxicity assessment

**DOI:** 10.1186/s40360-024-00826-7

**Published:** 2024-12-18

**Authors:** Emad Ali Albadawi, Eid Nassar Ali Musa, Hadel Mahroos Ghaban, Neven A. Ebrahim, Muayad Saud Albadrani, Ahmed I. El-Tokhy

**Affiliations:** 1 Department of Basic Medical Sciences, College of Medicine, Taibah University, Al-Madinah Al- Munawara, Saudi Arabia; 2 Department of Anatomy and Embryology, Faculty of Medicine, Cairo University, Cairo, Egypt; 3 Department of Human Anatomy and Embryology, Faculty of Medicine, Mansoura University, Mansoura, Egypt; 4 Department of Family and Community Medicine and Medical Education, College of Medicine, Taibah University, Al-Madinah Al-Munawara, Saudi Arabia; 5 Plant Protection Department, Faculty of Agriculture, Beni-Suef University, Beni-Suef, Egypt

**Keywords:** Silver nanoparticles (AgNPs), Nanocomposite, Green synthesis, Guava leaf extract, Organophosphorus insecticides, MTT assay

## Abstract

This study explores an eco-friendly approach to mitigate risks associated with organophosphorus insecticides, particularly Chlorpyrifos, by synthesizing silver nanoparticles (AgNPs) using Psidium guajava leaf extract and preparing a nanocomposite (AgNPs/S18) with Chlorpyrifos pesticide. The green-synthesized AgNPs and AgNPs/S18 nanocomposite were characterized using various analytical techniques, confirming the successful synthesis of AgNPs with an average size of 37 nm and forming a stable nanocomposite. Antibacterial assays demonstrated significant activity against Staphylococcus aureus, with AgNPs showing an 87.8% reduction and the nanocomposite achieving a 72% reduction in bacterial population. Cytotoxicity evaluations on normal liver and liver cancer cell lines revealed enhanced cytotoxicity of the nanocomposite compared to AgNPs alone, suggesting potential applications in targeted therapies. In vivo studies on rats revealed the protective effects of AgNPs and the nanocomposite against Chlorpyrifos-induced toxicity in liver and kidney tissues. Histopathological and ultrastructural analyses showed both treatments, particularly the nanocomposite, significantly mitigated cellular damage caused by Chlorpyrifos exposure. These findings suggest that green-synthesized AgNPs and their nanocomposite with Chlorpyrifos offer a promising approach to reducing pesticide hazards while maintaining efficacy. This research contributes to developing safer alternatives in pest management, addressing the need for more environmentally friendly agricultural practices while protecting human health and ecosystems.

**Supplementary Information:**

The online version contains supplementary material available at 10.1186/s40360-024-00826-7.

## Introduction

Pesticides are substances designed to control, repel, or eliminate pests that threaten crops, human health, or structures. These chemicals play a crucial role in current agriculture, contributing greatly to global food security and improved yields of crops [1]. Pesticides can be classified in several ways, including by their target organism, chemical structure, or mode of action. Common types include insecticides, herbicides, fungicides, rodenticides, bactericides, and nematicides [2]. Among these categories, insecticides form a crucial group, with organophosphates being one of the most significant classes. Organophosphate pesticides (OPs) were developed as an alternative to organochlorine pesticides in the 1940s. They are extensively utilized in agriculture, home environments, and “Public health programs for controlling mosquitoes [3].

OPs function by inhibiting the enzyme acetylcholinesterase (AChE), which is essential for the proper nervous system function in insects, mammals, and humans [4]. Chlorpyrifos, a widely used organophosphate insecticide globally, was first registered in the United States in 1965. It has been commonly used in agriculture to manage various pests on crops such as corn, soybeans, fruits, and vegetables. It is also used in non-agricultural settings such as golf courses and turf to control building pests [5]. The extensive use of Concerns over chlorpyrifos’ possible effects on human health, especially its neurotoxic qualities, have been raised [6, 7].

Many studies have connected exposure to chlorpyrifos during pregnancy and the early years of life to autistic spectrum disorders, attention deficit disorder (ADD), attention deficit hyperactivity disorder (ADHD), and developmental delays as well as lower IQ [8, 9]. These neurodevelopmental effects are particularly concerning as they can have long-lasting impacts on cognitive function and behavior. High-level exposure to Chlorpyrifos can cause acute poisoning symptoms, including nausea, dizziness, confusion, respiratory paralysis, and, in severe cases, death. These effects occur due to the inhibition of AChE, leading to an accumulation of acetylcholine in synapses [10].

Some research suggests that Chlorpyrifos may act as an endocrine disruptor, potentially interfering with hormonal systems and affecting reproductive health [11, 12]. Exposure has been linked to respiratory symptoms and reduced lung function, especially in agricultural workers and residents near treated fields [13, 14]. While the evidence is not conclusive, some studies have suggested a potential link between Chlorpyrifos exposure has been linked to certain types of cancer, such as “Lung cancer and non-Hodgkin’s lymphoma” [15, 16]. Emerging research suggests that exposure to chlorpyrifos may contribute to metabolic disorders such as obesity and diabetes, possibly through its effects on the gut microbiome and metabolic pathways [17, 18]. Due to health concerns, several countries have implemented restrictions or bans on using Chlorpyrifos. For instance, the European Union prohibited its use in 2020, and the United States Environmental Protection Agency (EPA) revoked all tolerances for Chlorpyrifos in 2021, effectively banning its use on food crops [19]. As concerns about the health effects of pesticides like Chlorpyrifos grow, researchers are exploring innovative approaches to mitigate their impact.

Green synthesis of metal and metal oxide nanoparticles has gained significant attention in recent years due to its eco-friendly nature and potential applications in various fields. These nanoparticles, including gold (AuNPs), silver (AgNPs), and palladium (PdNPs), have shown remarkable antioxidant, antibacterial, and photocatalytic properties. For instance, green-synthesized AuNPs using plant extracts have demonstrated potent antioxidant activities and significant antibacterial effects against human pathogens [20]. Similarly, AgNPs produced through green methods exhibit strong antibacterial effects against a wide range of pathogens, making them promising candidates for biomedical applications [21]. PdNPs synthesized using plant extracts have shown excellent catalytic activities and biological properties. A study by Basavegowda et al. [22] reported the successful synthesis of palladium nanoparticles using Saururus chinensis leaf extract. These PdNPs, with an average size of ~ 4 nm, exhibited moderate antioxidant activity (IC50: 26.66 µg/mL) and potent anti-tyrosinase activity (85.95% inhibition at 100 µg/mL). The eco-friendly synthesis method resulted in stable, spherical PdNPs with potential applications in catalysis, environmental remediation, and cosmetic industries [22]. The multi-functional nature of these green synthesized nanoparticles, combined with their reduced environmental impact, positions them as valuable tools in addressing various challenges in medicine, environmental science, and materials engineering.

Nanotechnology is manipulating matter at the nanoscale, usually involving particles of sizes between one and one hundred nanometers. Materials can display distinct qualities at this scale that set them apart from their bulk counterparts, creating new opportunities for various applications, such as pesticide management and environmental cleanup [23, 24]. Among the various types of nanoparticles, (AgNPs) have attracted particular interest in pesticide remediation and mitigation of health effects. AgNPs possess antimicrobial properties and are extensively utilized in medicine, water treatment, and consumer products [25]. In the context of pesticide management, AgNPs show promise in several ways. They have demonstrated the ability to catalyze the degradation of various pesticides, including organophosphates like Chlorpyrifos [26–28]. This catalytic activity is due to the high surface area and reactivity of AgNPs, which can accelerate the breakdown of pesticide molecules into less toxic compounds. The high surface area of (AgNPs) also enables effective adsorption of pesticide molecules, potentially reducing environmental exposure to pesticides by removing them from contaminated water or soil [29, 30]. Incorporating pesticides into nanoformulations with AgNPs may enhance their effectiveness while reducing pesticide use, leading to more targeted pest control with lower environmental impact [31, 32]. AgNPs have been found to potentially protect human cells and tissues from the dangerous effects of pesticides. This protective ability is linked to the antioxidant properties of AgNPs and their capacity to regulate cellular responses to oxidative stress [33, 34]. Nanoformulations incorporating AgNPs can be designed to manage the release of pesticides, possibly reducing how often they are used and lowering environmental pollution [35, 36]. Additionally, AgNPs can be functionalized to serve as sensitive and specific biosensors for detecting pesticide residues in environmental samples or biological specimens, aiding in monitoring and risk assessment efforts [37, 38]. To contextualize our research within the broader field of nanotechnology in agriculture, Table 1 provides an overview of various nanoparticles and their applications in mitigating pesticide hazards and promoting sustainable agricultural practices. This table highlights the diverse ways nanomaterials address challenges in modern agriculture, from enhancing pesticide efficacy and reducing environmental contamination to improving crop yield and stress tolerance. Our focus on silver nanoparticles (AgNPs) builds upon this foundation, exploring their potential to offer a more environmentally friendly approach to pest management while maintaining agricultural productivity.

**Table 1:** Applications of nanoparticles in pesticide hazard mitigation and sustainable agriculture

Nanoparticles type	Applications in pesticide hazard mitigation	Promotion of sustainable agriculture	References
Silver (Ag)	- Exhibits antimicrobial properties against plant pathogens - Enhances pesticide efficacy, potentially reducing the required dosage	- Reduces environmental contamination - Supports integrated pest management	[39–42]
Zinc Oxide (ZnO)	- Shows antifungal and antibacterial properties - Potential for slow release of pesticides	- Promotes plant growth - Enhances stress tolerance in crops	[43–47]
Titanium Dioxide (TiO₂)	- Photocatalytic degradation of pesticide residues - Antibacterial properties	- Enhances crop resistance to pathogens - Potential to improve nutrient uptake	[48–52]
Copper Oxide (CuO)	- Strong antibacterial and antifungal properties - Potential for controlled pesticide release	- May enhance plant disease resistance - Can act as a micronutrient source	[53–55]
Iron Oxide (Fe₂O₃)	- Antifungal properties - Potential for targeted pesticide delivery	- May improve plant stress tolerance - Can act as an iron source for plants	[56, 57]
Magnesium Oxide (MgO)	- Exhibits antibacterial properties - Potential for pesticide encapsulation	- May enhance plant growth - Can serve as a magnesium source	[58, 59]

Our research aims to investigate the potential of silver nanoparticles in reducing the harmful effects of organophosphate pesticides, with a specific focus on Chlorpyrifos. Our objectives include investigating the mechanisms by which AgNPs interact with Chlorpyrifos (organophosphate pesticides), evaluating the effectiveness of AgNPs in reducing Chlorpyrifos residues in contaminated water and soil samples, assessing the protective effects of AgNPs against Chlorpyrifos-induced toxicity in cellular and animal models, developing novel nanoformulations that combine AgNPs with Chlorpyrifos or alternative pesticides, exploring the use of functionalized AgNPs as biosensors, Assessing the possible risks linked to the use of silver nanoparticles in pesticide management, and investigating their potential to mitigate long-term health effects associated with chronic low-level exposure to Chlorpyrifos. We aim to contribute to developing safer and more sustainable pest management strategies to minimize the negative impacts of pesticides on human health and the environment. Integrating nanotechnology, specifically silver nanoparticles, with pesticide management shows promise in addressing the complex challenges of balancing agricultural productivity with environmental and public health concerns [60, 61]. It is important to remember that the potential of AgNPs in mitigating pesticide effects is promising, so further research is needed to understand their long-term impacts fully and develop practical, cost-effective applications. Our work aims to fill knowledge gaps and contribute to the developing field of nano-pesticide management. We strive to improve agricultural practices for safer and more sustainable outcomes, safeguarding the environment and human health.

## Materials and methods

Silver nitrate (AgNO_3_), Guava leaf extract (Psidium guajava), Distilled water (DW), S18 pesticide (Chlorpyrifos), Ethanol, and Filter paper. No additional purification was performed on any of the chemicals involved. This investigation prepared all solutions and rinses with pure (DW).

### Preparing of the guava *(Psidium guajava)* leaves extract

Guava (Psidium guajava) leaves were chosen for the green synthesis of silver nanoparticles due to their rich phytochemical profile, including flavonoids, phenolics, and terpenoids, which act as reducing agents and stabilizers in nanoparticle formation [62]. The widespread availability of guava and its leaves’ established antioxidant and antimicrobial properties make it an attractive option for eco-friendly nanoparticle synthesis [63, 64]. Recent studies have demonstrated the successful use of guava leaf extract in producing stable, uniform silver nanoparticles with beneficial properties. For instance, Rehab et al. (2019) [65] characterized silver nanoparticles synthesized using guava leaf extract, reporting an average size of 20–35 nm, while Wang et al. (2018) [62] demonstrated their potent antioxidant and antimicrobial activities. These findings support our choice of guava leaves for this research into green nanoparticle synthesis and applications. The leaves of the Guava plant, scientifically known as *Psidium guajava*, were cleaned with tap water after being obtained from a local marketplace. Subsequently, they were purified using water that had undergone a double distillation process to eliminate potential waste and impurities. Subsequently, they were left to undergo natural drying out for 5 days. The desiccated leaves were mixed with 200 mL of pure (DW) to prepare the leaf extract. The substance was subjected to a temperature of 65 degrees Celsius for 25 min. Subsequently, The extracts obtained from the leaves of the guava plant (Psidium guajava) underwent filtration and were then preserved for subsequent analysis [66, 67].

### Green synthesis of (AgNPs)

Prepare a solution of AgNO_3_ and dissolve 0.017 g in 100 mL of DW, resulting in a 1 mM concentration. Mix 50 mL of guava leaf extract with fifty mL of AgNO_3_ solution in a sterile beaker and rapidly agitate at ambient temperature. Observe the transition from transparent to dark brown color over time, indicating the transformation of Ag^+^ ions into Ag^0^ nanoparticles. Subject the solution to centrifugation at 15,000 revolutions per minute for 20 min to isolate pure silver nanoparticles. Separate the liquid from the top and combine the solid at the bottom with distilled water. Perform 2–3 consecutive washes. The presence of phytochemicals, specifically flavonoids and terpenoids, in guava leaf extract reduces Ag^+^ ions to Ag^0^ nanoparticles. Capping agents ensure nanoparticle stability by preventing aggregation [68, 69].

### Preparation of (AgNP/S18) nanocomposite

Mix 50 mL of guava extract with 50 mL of a 1-millimolar AgNO3 solution [70, 71]. Thoroughly mix the components and allow the reaction to occur overnight at room temperature. Centrifuge the mixture at a speed of 5000 revolutions per minute (RPM) for 10 min. to isolate the silver nanoparticles. Thoroughly wash the nanoparticles by immersing them in purified water and repeating the process 2–3 times. Ingest 50 mg of the previously produced silver nanoparticles. Use 100 mg of S18 insecticide. Dissolve in 10 mL of ethanol using an ultrasonic bath. Gradually introduce the S18 solution to the silver nanoparticles while exposing them to ultrasonication at 25 kHz. Apply sonication to the mixture for 30 min to enhance the attachment of S18 molecules to nanoparticles. Let the sonicated mixture stand overnight to allow the ethanol to evaporate. Collect the gray nanocomposite powder. The green reducing agent provides excellent control over the size of nanoparticles. The Sonochemical method facilitates the consistent adsorption of S18 onto silver nanoparticles, resulting in more extended release and improved effectiveness.

### Characterization

X-ray analysis was carried out using the EQUINOX 1000 instrument, manufactured by Thermo Scientific CO. in Lafayette, Colorado, United States of America, to ascertain the structure and phase identification of AgNPs produced through environmentally sustainable methods, as well as the AgNPs/S18 nanocomposite. The X-ray source was Cu Kα radiation, with a current of 31 milliamperes and a voltage of 33 kilovolts. The 2θ angles spanned from 5 to 80 degrees, with a scanning rate of 0.1 degrees per minute. The surface area and pore size of the AgNPs and AgNPs/S18 nanocomposite were measured using an N_2_ adsorption-desorption analyzer (Nova Touch 4 L, Quanta Chrome, Boynton Beach, Florida, United States of America) and the BET and DA techniques, respectively. Before the BET test, the AgNPs and AgNPs/S18 nanocomposite created from green sources were subjected to a degassing procedure at a temperature of 75 °C for 2 h to eliminate any moisture or gas molecules on their surfaces. The zeta seizer equipment NanoSight NS500, manufactured by Malvern Panalytical in Malvern, United Kingdom, was employed to measure the particle size and surface charge of the AgNPs and AgNPs/S18 nanocomposite created using environmentally friendly processes. The topographic properties of green-synthesized AgNPs and AgNPs/S18 nanocomposite were analyzed using AFM, SEM, and TEM tools. This facilitated the determination of their form and structure. The Agilent Technology AFM equipment, specifically the 5600LS model in Santa Clara, California, United States of America, was used to create 2D and 3D topographic images of the synthesized materials. Before the AFM examinations, the samples underwent a two-hour treatment with ultrasonic waves. The therapy was performed using an ultrasonic probe sonicator (UP400S, Hielscher, Oderstraße, Teltow, Germany) operating at a frequency of 59 kHz, an amplitude of 83%, and a cycle count of 0.79 for twenty minutes. A vacuum was used to form a thin coating of the samples using spin coater equipment (WS-650Sz, Laurell, North Wales, Pennsylvania, USA), working at 600 revolutions per minute. In addition, the Gwyddion software, developed by the Department of Nanometrology and Technical Length at the Czech Metrology Institute in Okružní, Czech Republic, was used to analyze the results obtained from the (AFM). The AFM pictures and data profiles were obtained using tapping mode imaging with a 100 nm × 67 nm wavelength. The utilized configurations were Al tap, 0.4 In/S speed, I. gain 0.4, and P. earn 20. The elemental analysis was conducted using a scanning electron microscope (JEOL, JSM-6701 F Plus, Peabody, Massachusetts, United States) with energy-dispersive X-ray spectroscopy (EDX). The green-synthesized AgNPs and AgNPs/S18 nanocomposite were examined using TEM (JEOL, JEM-2100 high-resolution, Peabody, Massachusetts, United States) to gather data on their dimensions, configuration, and surface structure. SEM was used to capture the photos, employing an acceleration voltage of 10 kV and a magnification of 3000 times (3000 Kx). The AgNPs and AgNPs/S18 nanocomposite were combined with double-distilled water and subjected to sonication for twenty minutes using an ultrasonic probe sonicator operating at 55 kHz, an amplitude of 55%, and a cycle of 0.55. This was conducted before the examination using TEM. After depositing droplets of the dispersed mixture, which varied in size from 5 to 10 microns, onto a carbon-coated copper grid, we conducted a TEM investigation.

### In-vitro study

#### Colony forming unit counting test (CFU)

The antimicrobial properties of the AgNPs and nanocomposite were evaluated against *Staphylococcus aureus (S. aureus)* utilizing the agar plating technique. An *S. aureus* suspension was prepared according to the McFarland 0.5 standard and cultured in Mueller-Hinton broth to assess antimicrobial efficacy. The experiment involved adding 200 µL of the bacterial suspension to a 96-well plate containing test samples and controls (DMSO). This plate was then kept at 37 °C for 24 h. Following this incubation, 20 µL of the resulting bacterial solution was spread onto pre-dried nutrient agar plates, which were subsequently incubated for an additional 24 h at 37 °C [72]. The resulting bacterial colonies were visually documented using a digital camera, and a colony count was performed. The formula for calculating antimicrobial effectiveness was as follows:

$$\begin{aligned}&\mathbf{T}\mathbf{h}\mathbf{e}\:\mathbf{a}\mathbf{n}\mathbf{t}\mathbf{i}\mathbf{b}\mathbf{a}\mathbf{c}\mathbf{t}\mathbf{e}\mathbf{r}\mathbf{i}\mathbf{a}\mathbf{l}\:\mathbf{r}\mathbf{a}\mathbf{t}\mathbf{i}\mathbf{o}\:\left(\mathbf{\%}\right)\cr&\quad=\frac{\left(\begin{aligned}&\mathbf{N}\mathbf{u}\mathbf{m}\mathbf{b}\mathbf{e}\mathbf{r}\:\mathbf{o}\mathbf{f}\:\mathbf{C}\mathbf{F}\mathbf{U}\mathbf{s}\:\mathbf{i}\mathbf{n}\:\mathbf{t}\mathbf{h}\mathbf{e}\:\mathbf{c}\mathbf{o}\mathbf{n}\mathbf{t}\mathbf{r}\mathbf{o}\mathbf{l}\:\mathbf{g}\mathbf{r}\mathbf{o}\mathbf{u}\mathbf{p}\cr&\quad-\mathbf{N}\mathbf{u}\mathbf{m}\mathbf{b}\mathbf{e}\mathbf{r}\:\mathbf{o}\mathbf{f}\:\mathbf{C}\mathbf{F}\mathbf{U}\mathbf{s}\:\mathbf{i}\mathbf{n}\:\mathbf{t}\mathbf{h}\mathbf{e}\:\mathbf{e}\mathbf{x}\mathbf{p}\mathbf{e}\mathbf{r}\mathbf{i}\mathbf{m}\mathbf{e}\mathbf{n}\mathbf{t}\mathbf{a}\mathbf{l}\:\mathbf{g}\mathbf{r}\mathbf{o}\mathbf{u}\mathbf{p}\end{aligned}\right)\times\:100}{\left(\mathbf{N}\mathbf{u}\mathbf{m}\mathbf{b}\mathbf{e}\mathbf{r}\:\mathbf{o}\mathbf{f}\:\mathbf{C}\mathbf{F}\mathbf{U}\mathbf{s}\:\mathbf{i}\mathbf{n}\:\mathbf{t}\mathbf{h}\mathbf{e}\:\mathbf{c}\mathbf{o}\mathbf{n}\mathbf{t}\mathbf{r}\mathbf{o}\mathbf{l}\:\mathbf{g}\mathbf{r}\mathbf{o}\mathbf{u}\mathbf{p}\right)}\end{aligned}$$

#### MTT assay

The MTT assay is a widely adopted colorimetric method for evaluating cellular metabolic activity, which indicates cell viability and cytotoxicity. This protocol delineates a standardized approach to assess the cytotoxic impact of AgNPs and nanocomposite on cultured THLE-2 cells using the MTT (3-(4,5-dimethylthiazol-2-yl)-2,5-diphenyltetrazolium bromide) tetrazolium reduction test. The procedure measures the cells’ ability to convert the yellow MTT compound into purple formazan crystals, providing insights into metabolic function and overall cell health when exposed to potentially toxic substances [73]. Inoculate a 96-well tissue culture plate with 1 × 10^5^ cells/mL (100 µL/well) and incubate at 37 °C for 24 h to form a complete monolayer. After achieving confluence, decant the growth medium and wash the cell monolayer twice with wash media. Prepare two-fold dilutions of the test sample in RPMI medium with 2% serum, then apply 0.1 mL of each dilution to separate wells, leaving 3 wells as controls with only maintenance medium. Incubate the plate at 37 °C and monitor cells for signs of toxicity. Prepare MTT solution (5 mg/mL in PBS) using BIO BASIC CANADA INC reagent. Add 20 µL MTT solution to each well, shake at 150 rpm for 5 minutes to mix, then incubate at 37 °C, 5% CO_2_ for 4 h. Remove the media and dry the plate if necessary. Add 200 µL DMSO to each well to resuspend formazan, shaking at 150 rpm for 5 minutes to mix thoroughly. Finally, record optical density at 560 nm and subtract background at 620 nm. The optical density should directly correlate with cell quantity [74, 75].

### In vivo study

#### Animal

The study was conducted on 40 adult male Sprague Dawley albino rats weighing between 200 and 250 g and aged between 12 and 14 weeks. The rats were obtained from the Animal House at Taibah University in Saudi Arabia. Before any acts took place, the animals were kept in controlled laboratory conditions for 14 days. They were granted unrestricted access to water and food. The participants were housed in well-ventilated enclosures, alternating between darkness and light daily.

#### Research methodology

*Rats were classified into 4 groups*:

Group I: Control group (no treatment). Group II: Blank silver nanoparticles (AgNPs) by oral route. Group III: Organophosphorus pesticide S18 by oral route. Group IV: AgNPs/S18 nanocomposite by oral route. Afterwards, blood was collected, and then animals were sacrificed via cervical dislocation. The treatment dose was 5 mg/kg, administered orally once daily for 28 days [76, 77].

#### Biological assays

Blood was collected from anaesthetized rats by extracting it from the retro-orbital plexus. The blood was then kept on ice until centrifuged at ambient temperature for 15 min at a force of 1500 g. After centrifugation, the serum was obtained and stored at -80 °C until it was ready for further analysis. The levels of liver enzymes were measured in the samples [78–80].

#### Collection and processing of tissue samples

The liver tissue of rats was rapidly extracted and dissected, followed by rinsing to eliminate any remaining blood using a chilled solution of 0.9% saline. The right lobe was preserved in 10% formalin and subsequently embedded in paraffin blocks for histological analysis, with sections cut at a thickness of 4 μm. Meanwhile, the left lobe was homogenized using the SR30 model PS 80 in 5–10 mL of PBS (0.1 M, pH 7.4). The suspension underwent two freeze-thaw cycles, followed by cold centrifugation of the homogenates at 4 °C for 15 min at 10,000 g. The liquid portion was gathered, separated, and preserved at -80 °C for molecular and biochemical examinations [81].

#### Light microscopic examination

The liver and kidney were prepared for investigation under a light microscope. The tissue samples were prepared and processed using hematoxylin and eosin (H and E) stains. The specimens were analyzed using a light microscope [82].

#### Transmission electron microscopic examination (TEM)

TEM was utilized to examine the ultrastructural impacts of pesticides, AgNPs, and nanocomposites on kidney and liver tissues [83, 84].

#### Statistical analysis

The data were subjected to statistical analysis using the “One-way ANOVA test.” The values were reported as the mean ± standard deviation. A statistical significance threshold of *p* < 0.05 was considered significant.

## Results and discussion

### Characterization of eco-friendly synthesized AgNPs and AgNP/S18 nanocomposite

#### UV-Vis spectral analysis

**Fig. 1:**
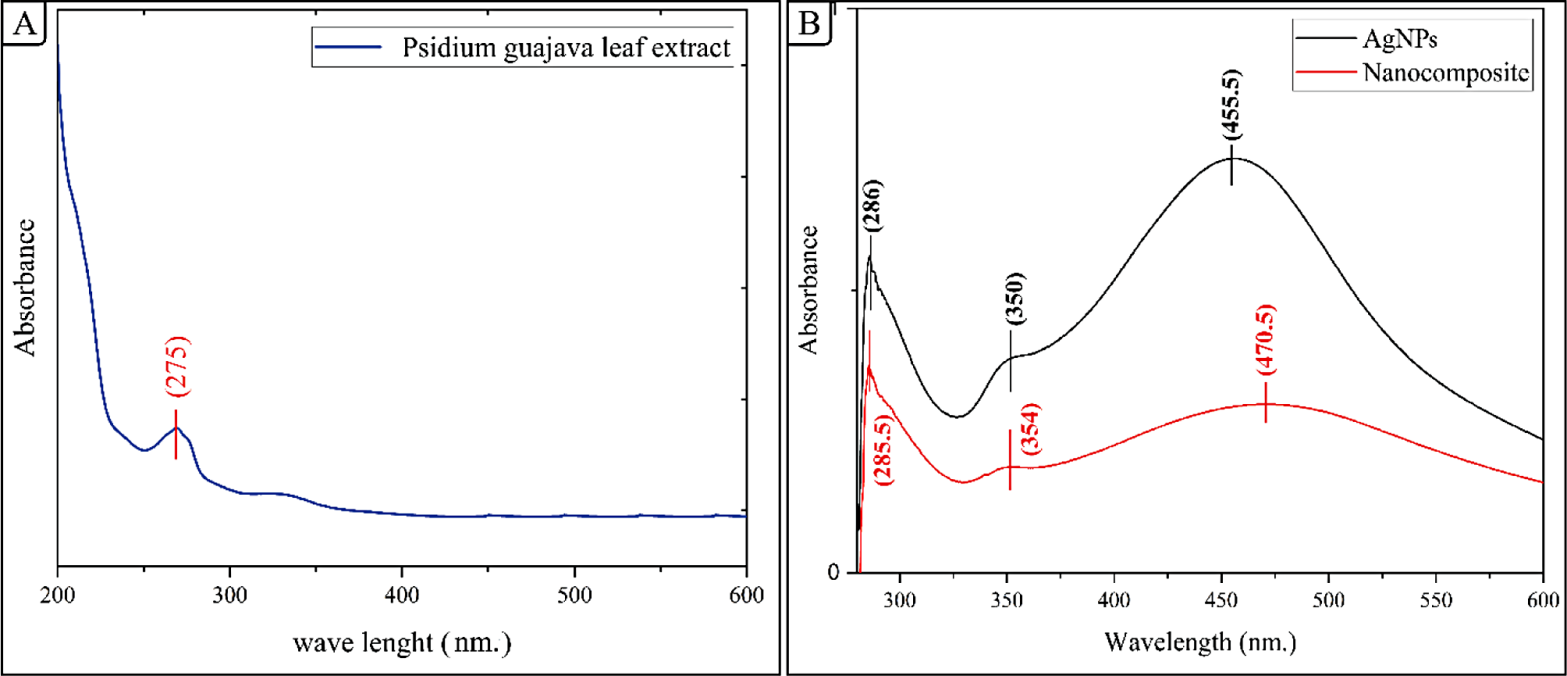
UV-Vis Spectra of **A** (Psidium guajava Leaf Extract) and **B** (AgNPs and AgNP/S18 Nanocomposite)

*Psidium guajava* leaves contain a high concentration of alkaloids, phenol, flavonoids, phytosterols, and terpenoids [85]. The water-soluble constituents of *Psidium guajava* leaves facilitated the transformation of Ag^+^ ions to Ag^0^ nanoparticles. Capping agents enhance stability by preventing the aggregation of nanoparticles [86]. The *Psidium guajava* leaves extract, and AgNO_3_ were combined and incubated at ambient temp for 4 h. The UV-visible spectrum of Psidium guajava leaf extract from 200 to 600 nm shows characteristic absorption patterns of plant phytochemicals. A strong peak below 275 nm indicates aromatic amino acids and conjugated systems. A shoulder around 280–290 nm suggests phenolic compounds like flavonoids. Absorption decreases with increasing wavelength, with a slight plateau at 300–350 nm, possibly from flavonoids or phenolic acids. Low, constant absorption beyond 400 nm indicates minimal visible light absorption. This profile characterizes the extract’s composition and potential biological activities, as shown in Fig. 1(A). The creation of silver nanoparticles was indicated by a transition in hue from colorless to reddish brown. The reaction mixture underwent centrifugation at a speed of 5000 RPM for ten minutes to separate and obtain the silver nanoparticles that were produced (AgNPs). UV-visible analysis is a highly effective method for characterizing AgNPs. The UV-Vis spectra of noble metal nanoparticles exhibit distinct absorption peaks due to the interaction between surface electrons and light photons, resulting in sharp surface plasmon resonance [87]. The dimensions, configuration, and dispersion of nanoparticles dictate the properties of each metal. The absorption peaks at 350 nm and 275 nm are characteristic of flavonoids and phenols, respectively. These peaks are attributed to n – π* and π – π* transitions [88]. Additionally, these peaks show the presence of an amino acid that plays a role in the reduction and stability of AgNPs. The UV-Vis absorption spectra of the AgNPs synthesized using *Psidium guajava* leaf extract demonstrate a plasmon resonance band at 454 nm, as observed in Fig. 1(B) [89, 90]. This indicates the formation of AgNPs. It is worth noting that the UV-Vis spectrum of the leaf extract itself was also tested, but no peak was observed in the range of 280–800 nm. Prior research has indicated the presence of a peak in the surface plasmon resonance (SPR) spectrum of AgNPs at wavelengths ranging from 410 to 450 nm. The presence of spherical nanoparticles likely causes this peak.

#### FTIR spectral analysis

**Fig. 2:**
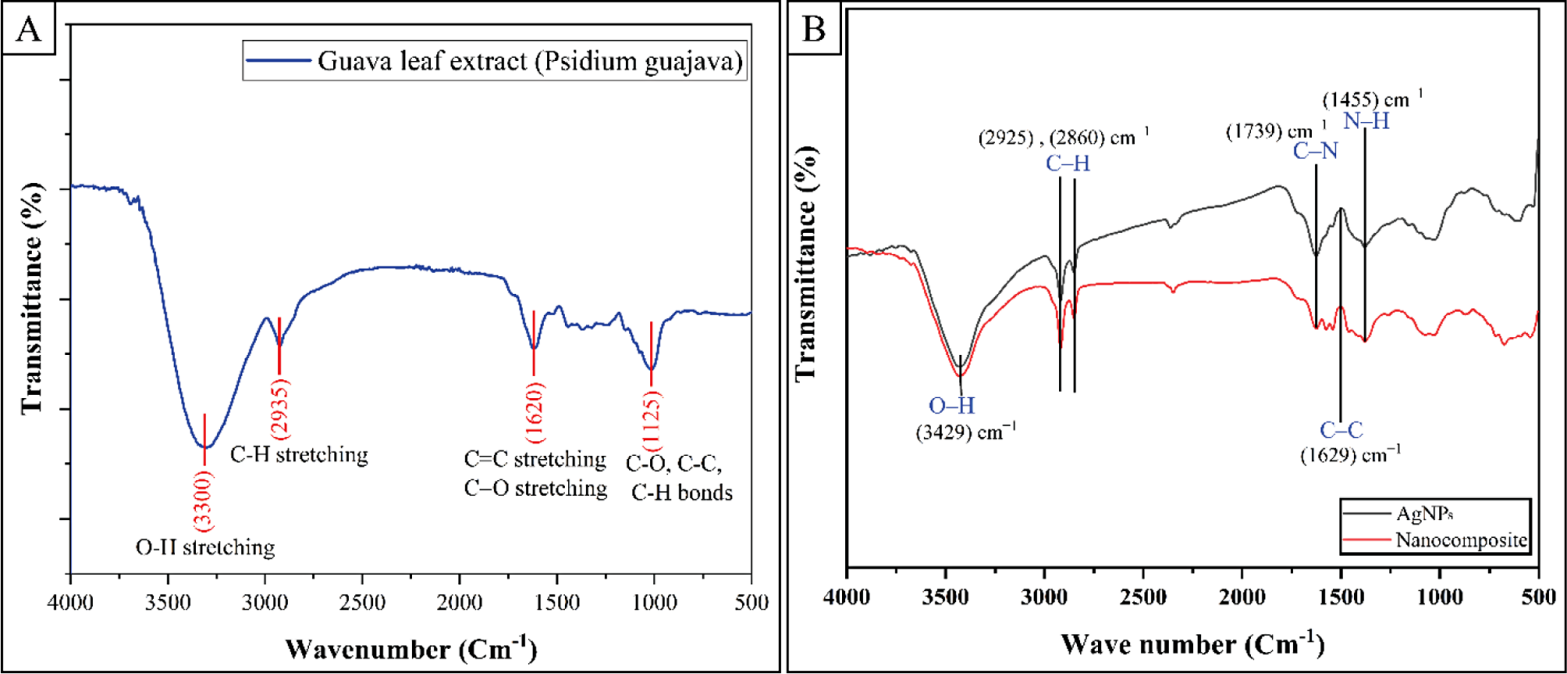
FT-IR spectra of **A** (Psidium guajava Leaf Extract) and **B** (AgNPs and AgNPs/S18 Nanocomposite)

FT-IR spectroscopy was performed to identify several functional groups in biomolecules responsible for the biological reduction of Ag^+^ and the capping/stabilization of AgNPs. The intensity of the bands was compared to standard values to ascertain the functional categories. The IR spectrum of guava leaf extract (4000–500 cm⁻¹) reveals key chemical components (Fig. 2(A)). A broad band at 3400–3200 cm⁻¹ indicates O-H stretching from phenolics and hydroxyl-containing molecules. Weak absorption at 3000–2800 cm⁻¹ suggests C-H stretching of alkyl groups. A peak at 1600 cm⁻¹ may be from C = C or C = O stretching. The fingerprint region below 1500 cm⁻¹ shows complex patterns typical of diverse plant compounds, with notable peaks at 1400, 1200, and 1000 cm⁻¹. This spectral profile provides a molecular fingerprint of the extract’s functional groups and potential bioactive components, as shown in Fig. 2(A).

The FT-IR spectrum, shown in Fig. 2(B), displays absorption peaks at 3429, 2925, 2860, 1739, 1629, 1455, 1378, 1243, 1041, and 594 cm^− 1^, indicating the presence of a capping agent in addition to the AgNPs [91, 92].

The bands seen at 3429 cm^− 1^ in the spectra correspond to the stretching vibration of the O-H bond, which indicates the presence of alcohol and phenol. Band observations were conducted in the range of 2925 and 2860 cm^− 1^, associated with the elongation of C-H bonds in aromatic compounds. The band detected at a wavenumber of 1739 cm^− 1^ was ascribed to the elongation of carbon-carbon bonds that are not conjugated. The peak detected at 1629 cm^− 1^ in the spectra corresponds to the stretching vibrations of the C-N and C-C bonds, suggesting the existence of proteins. The band detected at a wavenumber of 1455 cm^− 1^ corresponds to the stretching vibration of the N-H bond, which is present in the amide bonds of proteins. These functional groups have been shown to contribute to the stability and capping of AgNPs in multiple investigations [93]. The bands at 1450 cm^− 1^ and 1041 cm^− 1^ were identified as the proteins’ N-H and C-N (amines) stretch vibrations, respectively [94]. The band at a wavenumber of 1378 cm^− 1^ demonstrates the NO symmetry stretching commonly found in nitro compounds [95]. The band seen at 1243 cm^− 1^ corresponds to the elongation of the C–N bond in amines. The occurrence of a band in the 594 cm^− 1^ range can be attributed to the elongation of the C-Br bond, a characteristic of alkyl halides. The FT-IR spectroscopy investigation reveals that the presence of Ag^+^ ions or nanoparticles in *Psidium guajava* leaves does not alter the secondary structure of proteins.

#### XRD

**Fig. 3:**
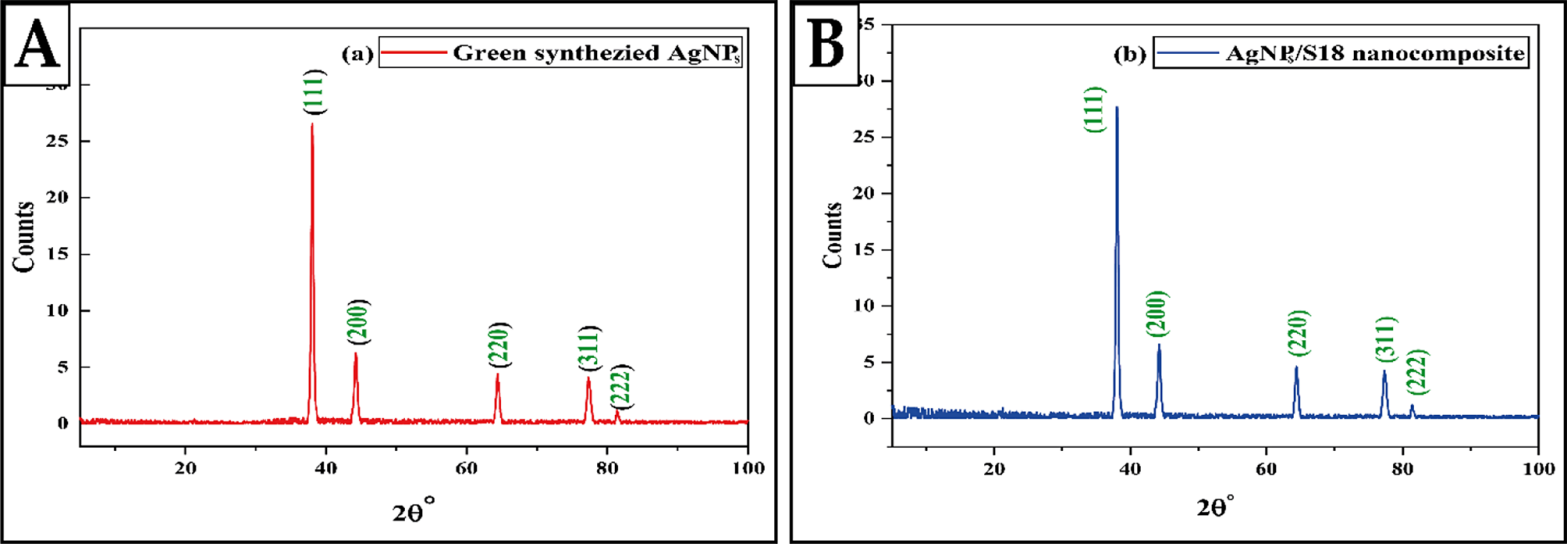
XRD Patterns of Synthesized **A** AgNPs and **B** AgNPs/S18 Nanocomposite

The crystalline nature of the nanoparticles was confirmed using X-ray crystallography. Figure 3(A and B) displays the XRD pattern of the produced AgNPs and nanocomposite. The reported intensities were obtained using diffraction in the 20 to 80°. The presence of five prominent Bragg reflections at angles of 38.12°, 44.33°, 64.45°, 77.41°, and 81.56° can be attributed to the planes with Miller indices **(1 1 1)**,** (2 0 0)**,** (2 2 0)**,** (3 1 1)**,** and (2 2 2)** respectively [96]. These planes correspond to a face-centered cubic crystal structure characteristic of silver. The computed interplanar spacing (d) values for the **(1 1 1)**, **(2 0 0)**, **(2 2 0)**, **(3 1 1)**, and **(2 2 2)** planes are 2.36, 2.04, 1.44, 1.23, and 1.18 Å, respectively [95]. These values have been compared to the typical silver nanoparticle values. The average size of the crystals is determined by employing the Debye-Scherrer formula.

$${\bf{\it{D}}}\,{\bf{\it{ = }}}\,{\bf{{\it k\lambda}{\beta cos\theta}}}$$

The average size of the nanoparticles is denoted by D and is measured in terms of crystalline structure. The geometric factor is denoted by k (with a value of 0.9), the wavelength of the X-ray radiation source is λ (with a value of 0.15418), and β represents the angular full-width at half maximum (FWHM) of the XRD peak at the diffraction angle θ (Dubey et al., 2010). The average crystallite size of the AgNPs is around 9.038 nm, as shown in Table 2.

**Table 2:** Structural parameters of synthesized leaf extract-mediated AgNP

Miller Indices (hkl)	Interplanar Spacing (d)	Bragg Angle (2θ)	FWHM (β)	Lattice Parameter (a) Å	Crystallite size (D) nm
(1 1 1)	2.36 Å	38.12°	0.79	4.086	11.12
(2 0 0)	2.04 Å	44.33°	0.92	4.078	9.74
(2 2 0)	1.44 Å	64.45°	1.14	4.086	8.61
(3 1 1)	1.23 Å	77.41°	1.34	4.086	7.94
(2 2 2)	1.18 Å	81.56°	1.41	4.090	7.78

#### BET surface area and porosity properties

**Fig. 4:**
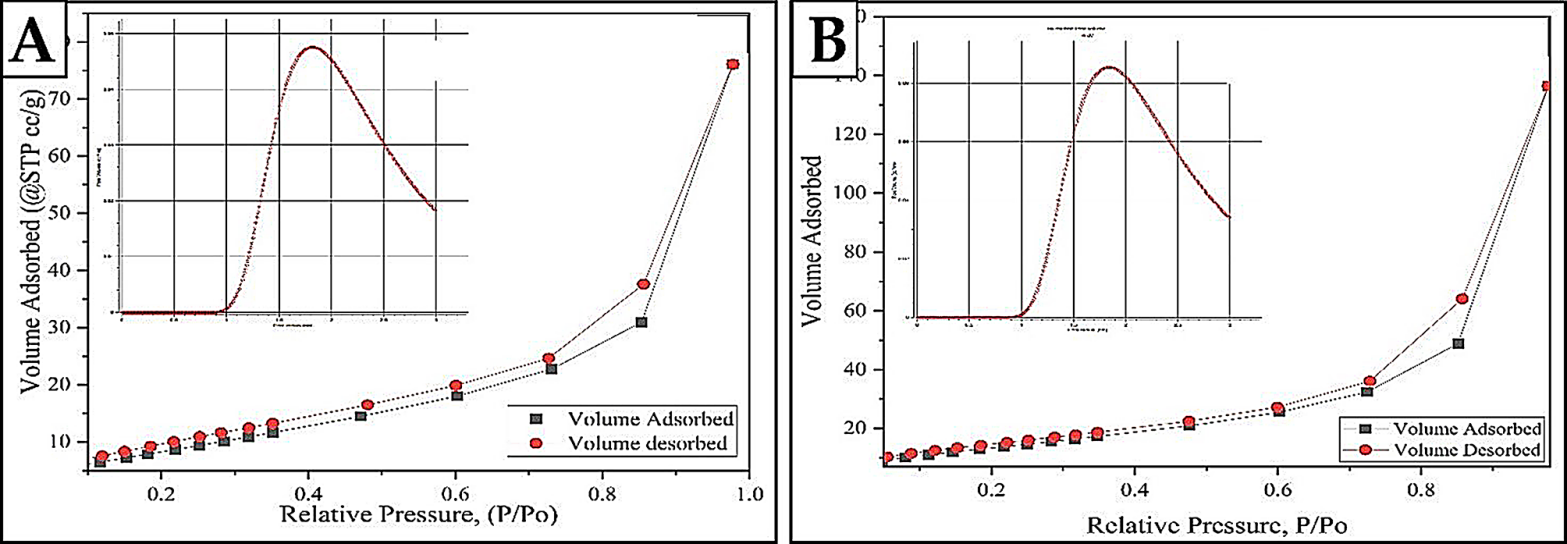
BET surface area and porosity analysis of **A** AgNPs and **B** AgNPs/S18 Nanocomposite shows the N_2_ adsorption-desorption isotherm curves using the BET method and the pore size/volume calculated by the DA method for both materials

Examining BET surface area and porosity enhances imaging and other bulk approaches by offering crucial insights into the internal surface features of a material [97]. It is vital to uncover the connections between the structure and its function when working with nanoporous designs. The adsorption-desorption isotherm curves of N_2_ for both AgNPs and AgNPs/S18 nanocomposite, produced using an environmentally sustainable method and evaluated using the BET approach, can be seen in Fig. 4(A and B). The pore size distributions of Green-synthesized AgNPs and AgNPs/S18 nanocomposites are also illustrated in Fig. 4(A and B), respectively. Based on the IUPAC categorization of adsorption isotherms, the isotherm curve of AgNPs falls into the type III category [97, 98]. The absence of a discernible “sharp knee” shape suggests that the interactions between adsorbates are more substantial than between adsorbates and the adsorbent. De Boer’s taxonomy of hysteresis loops categorizes the green AgNPs that were created as exhibiting H3 hysteresis, which is characterized by wedge-shaped holes. The isotherm curve of the AgNPs/S18 nanocomposite displays a type IV adsorption isotherm, suggesting that the adsorbents used are either non-porous or microporous, and show unrestricted adsorption of a monolayer or several layers, respectively [99–101]. This isotherm demonstrates that the amount of adsorption rises at low relative pressures within a short period. This occurs when the adsorbate molecules initially come into contact with the region of more incredible energy and subsequently interact with the lower energy region. After the molecules have formed a monolayer, the construction of multilayers happens when the isotherm reaches a “sharp knee” and the relative pressure approaches one. At this point, there is a significant increase in signal, indicating that the adsorbate gas is starting to condense into a liquid. An H2 hysteresis phenomenon was observed in the AgNPs/S18 nanocomposite. This phenomenon was ascribed to inkbottle-shaped cavities linked to capillary condensation mechanisms in mesoporous materials. This hysteresis loop provides evidence of the existence of complex pore networks. Table 3 presents a concise summary of the findings regarding the area, volume, and pore size. These results confirmed that the pesticide loaded on a green synthesized silver nanoparticle surface.

**Table 3:** Summary of the BET surface area, volume, and pore size results of AgNPs and nanocomposite

Nano-particle /Nanocomposite	Total Pore Volume (cc/g)	Surface Area (SBET) (m^2^/g)	Average Micropore Size (nm) (DA Method)	Average Pore Size (nm)
AgNPs	0.211674	50.7563	0.149	8.3408
AgNPs/S18 nanocomposite	0.118046	35.0007	0.088	6.74532

#### Zeta potential and DLS

**Fig. 5:**
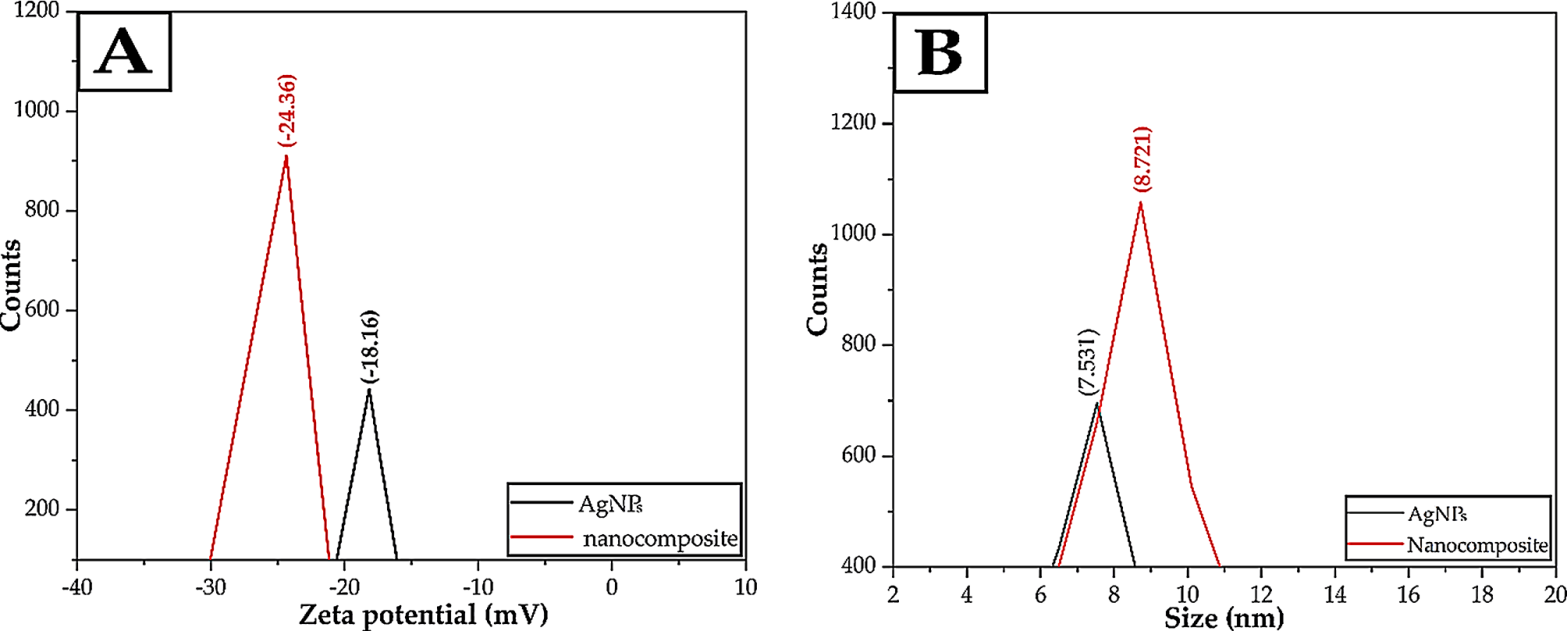
**A** The zeta potential pattern is displayed for the green synthesized AgNPs and the AgNPs/S18 nanocomposite. **B** The *DLS* pattern is displayed for the green synthesized AgNPs and the AgNPs/S18

DLS provides quick particle size measurements for monitoring stability [102, 103], while zeta potential predicts future issues with dispersion or aggregation. This information is crucial for producing nanomaterials, understanding biological interactions, and various commercial uses. The particle size of the generated AgNPs and AgNPs/S18 nanocomposite was evaluated using DLS techniques. The measurements were conducted using eco-friendly protocols. The mean dimensions of the AgNPs synthesized using green methods and the AgNPs/S18 nanocomposite were 7.531 and 8.721 nm, respectively, as depicted in Fig. 5(B). All suspensions in colloidal form demonstrated exceptional stability and maintained uniform sizes. The investigation revealed that the size distribution exhibited an unimodal distribution with polydispersity indices. The enhanced stability of the AgNPs with the S18 insecticide is demonstrated by the augmentation in the mean size of the nanocomposite consisting of AgNPs and S18. The average size refers to a hydrodynamic measurement that considers both nanoparticles and solvent molecules associated with the particle in motion. This work aims to analyze the stability of the AgNPs and AgNPs/S18 nanocomposite in water-based solutions by measuring the zeta potentials of these nanomaterials at different voltage levels, as shown in Fig. 5(A). The voltage values of the green-produced AgNPs and the AgNPs/S18 nanocomposite were measured to be -18.16 mV and − 24.36 mV, respectively [104, 105].

#### SEM and TEM

**Fig. 6:**
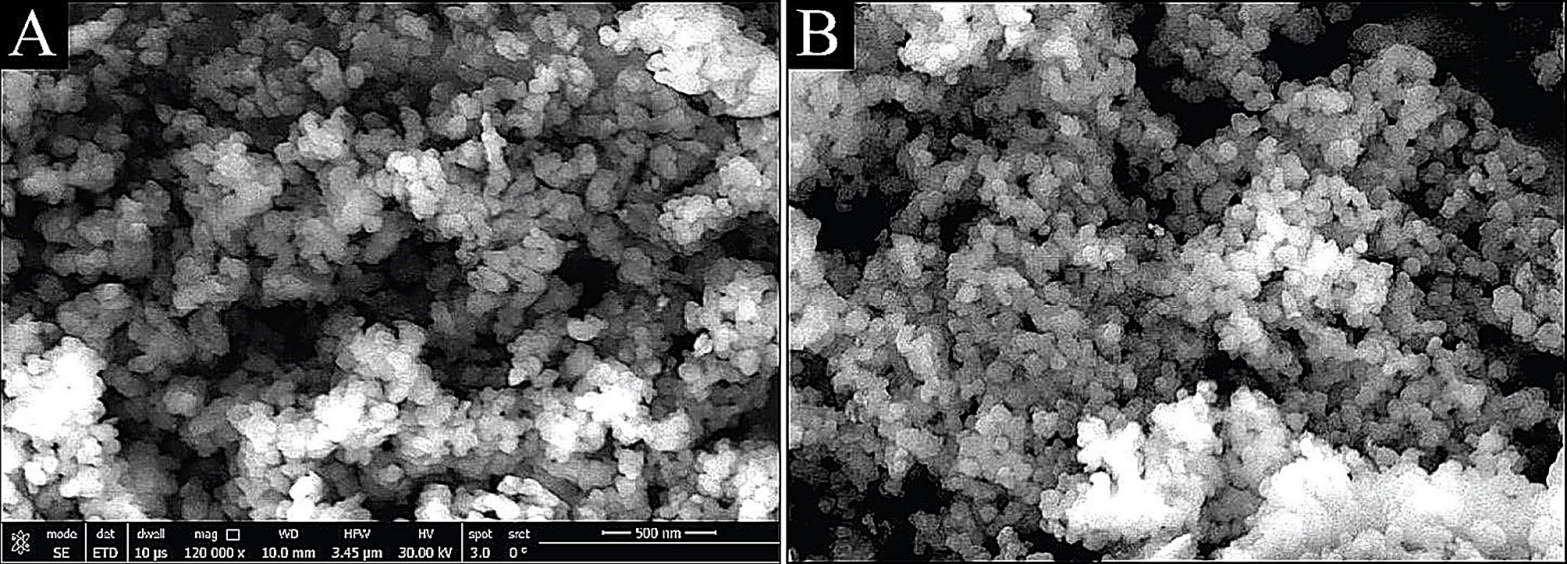
SEM Micrographs of **A** Green synthesized AgNPs and **B** AgNPs/S18 nanocomposite

Figure 6(A and B) display scanning electron micrographs (SEM) of green synthesized AgNPs and AgNPs/S18 nanocomposite, respectively [106]. According to the image of AgNPs, the particles were organized in rectangle bipyramid objects and showed a high crystallinity level. This can be seen in the picture. Additionally, the particles developed independently, with diameters ranging from 100 nm, and they were distributed in a monodisperse fashion [93, 107].

**Fig. 7:**
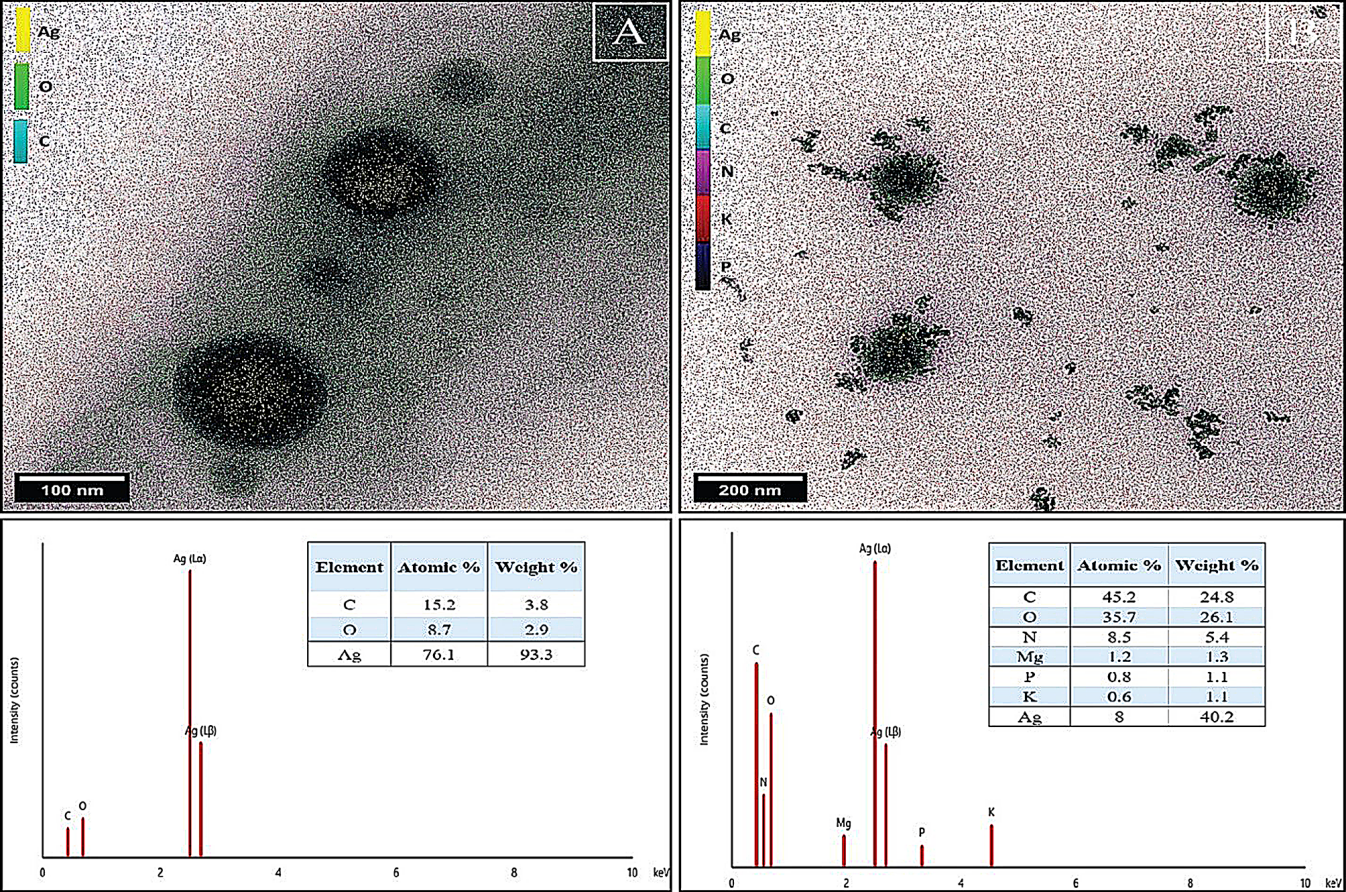
TEM and STEM-EDS analysis where **A** TEM image and elemental mapping of green synthesized AgNPs and **B** TEM image and elemental mapping of AgNPs/S18 nanocomposite

These findings further support the success of the environmentally friendly manufacture of AgNPs utilizing guava (*Psidium guajava*) leaf extract. Furthermore, the picture of the AgNPs/S18 nanocomposite displayed a distinct texture, which can be related to the characteristics of the AgNPs. An additional validation of the dispersity of the particles that were manufactured was supplied by the (TEM) image of green AgNPs. The TEM image Fig. 7(A) (an elemental mapping image of the AgNPs obtained through STEM-EDS) showed that the individual particles formed in spherical morphology with uniform distribution structures with a size of around 37 nanometers [94, 108], which refers to the range of particle sizes from 30 to 40 nanometers, on the other hand, a few dots represent the biomolecules that serve as reducing agents and cap the surfaces of the nanoparticles, so preventing them from aggregating. Studies that were very similar to this one demonstrated that a comparative plant extract can produce symmetrical nanoparticles. Spherical in shape, the composite of silver and insecticide had a mean diameter of approximately sixty nanometers.

It is possible to witness the adhesion of organophosphorus on the surface of green-produced AgNPs, which reveals the successful development of a nanopesticide composite with uniform distribution. This is evident in the inset of Fig. 7(B). Figure 7(B) shows the typical TEM image of the resulting Ag/pesticide composite. The Ag/pesticide composite was spherical, with a mean diameter of about 60 nm. From the inset of Fig. 7(B) (an elemental mapping image of the AgNPs/S18 nanocomposite obtained through STEM-EDS), the adhering of organophosphorus on the surface of green synthesized AgNPs could be observed, revealing the successful formation of nanopesticide composite with homogenous distribution.

The accompanying elemental analysis tables provide quantitative compositional data. For the AgNPs (Fig. 7A), silver dominates at 76.1 atomic% and 93.3 weight, with minor carbon and oxygen content from capping agents. In contrast, the nanocomposite (Fig. 7B) shows increased carbon and oxygen levels (45.2% and 35.7% atomic, respectively) and the presence of Mg, P, and K. This compositional change supports the successful formation of the AgNPs/S18 nanocomposite. The TEM results align well with SEM findings, confirming the spherical morphology and size distribution of AgNPs and AgNPs/S18 nanocomposite while providing additional elemental information that validates the incorporation of the organophosphorus pesticide onto the AgNP surface.

#### AFM

**Fig. 8:**
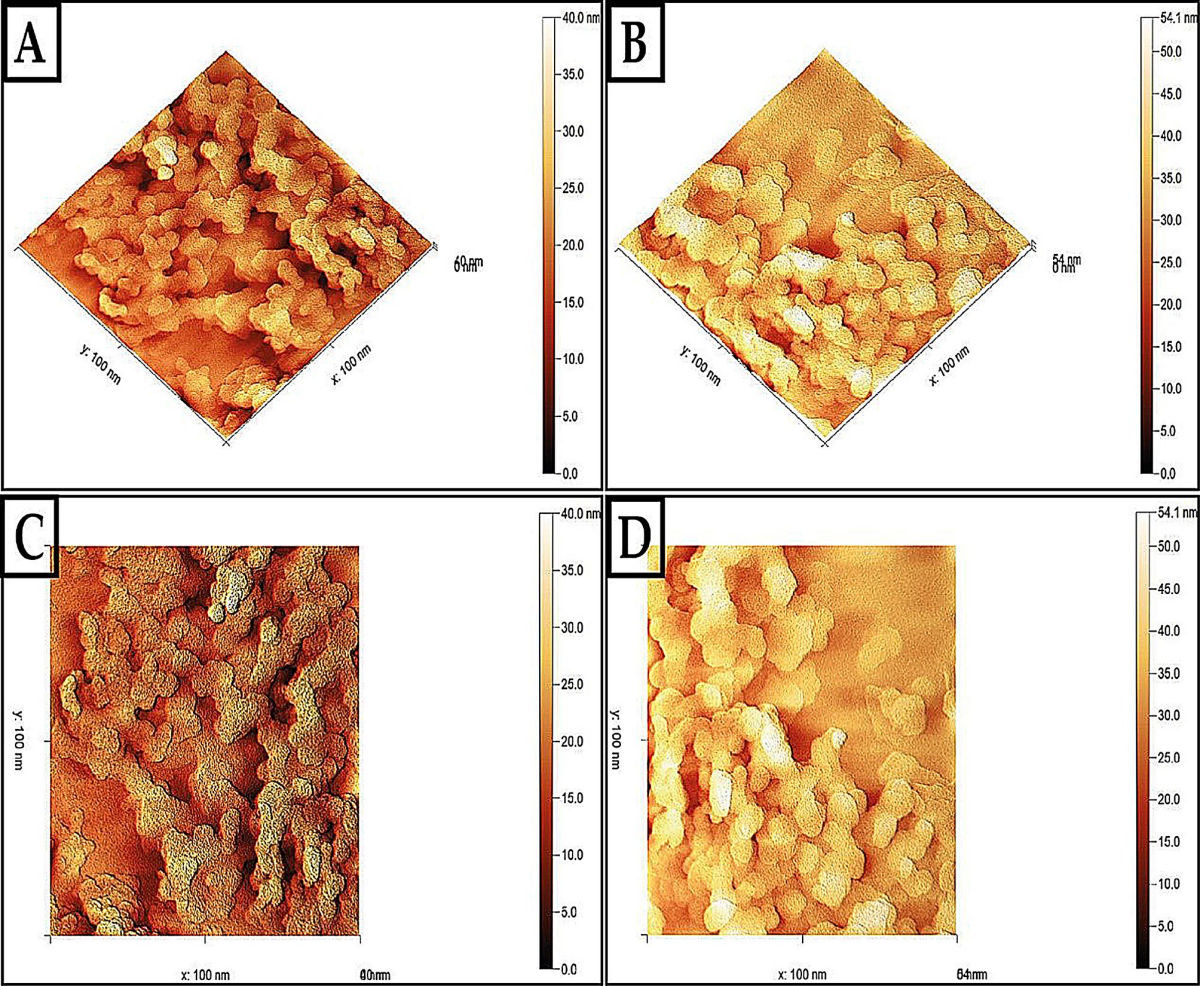
**A** 3D AFM images of AgNPs. **B** 3D AFM images of AgNPs and AgNPs/S18 nanocomposite. **C** 2D AFM images of AgNPs. **D** 2D AFM images of AgNPs/S18 nanocomposite

Using eco-friendly methods, the atomic force microscope (AFM) was utilized to examine the surface topography of the AgNPs and AgNPs/S18 nanocomposite [109]. Figure 8(C and D) displays the two-dimensional image of green-produced AgNPs and AgNPs/S18 nanocomposite, respectively. In contrast, Fig. 8(A and B) shows the three-dimensional image of AgNPs AgNPs/S18 nanocomposite. While certain particles exhibited modest variations in size and shape and had some overlaps, the homogeneity and uniformity of AgNPs were observable. The green-synthesized AgNPs had rhombus bipyramid forms and were produced with excellent crystallinity and sharp edges. The nanoparticles are inside the 100 nm scale, with the surface height measuring 61.2 nm [110, 111]. This results in the granular border moving more freely, which leads to an increase in granule sizes and a reduction in internal and surface flaws in the structural texture. This also results in a strong cohesion among the granular boundaries. The mechanical, electrical, and magnetic properties are enhanced. The nanocomposite’s surface displayed needle-like forms, and its height grew to 92.7 nm. The results align with the porosity data obtained from the BET analysis. The average grain size determined from AFM pictures is greater than the particle sizes measured with SEM and TEM, suggesting that each grain results from the aggregation of several nanocrystals.

#### Mechanism of action of AgNPs in pesticide hazard mitigation

**Fig. 9:**
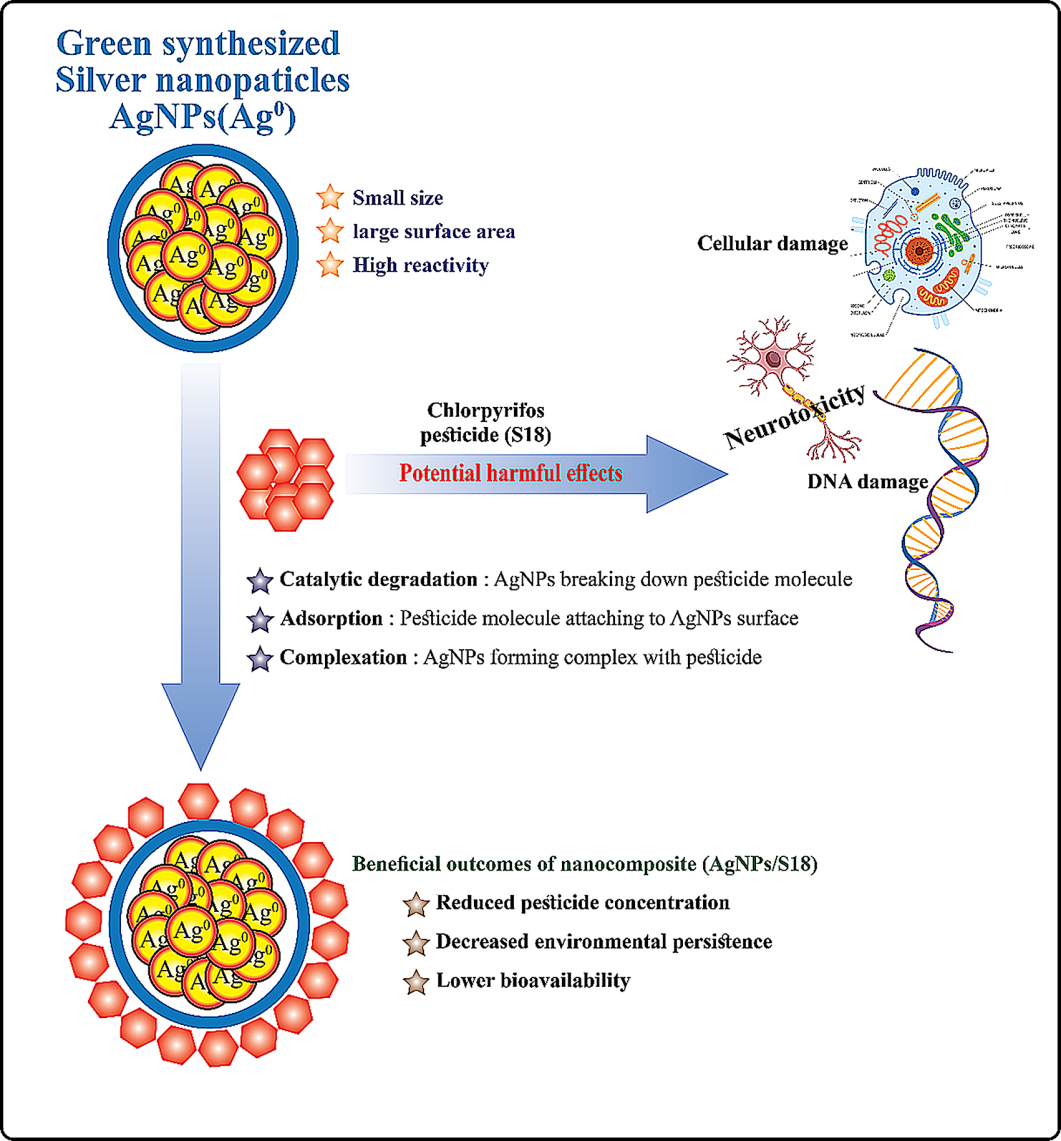
Schematic representation of green-synthesized silver nanoparticles (AgNPs) interaction with chlorpyrifos pesticide (S18) and the resulting nanocomposite effects

Figure 9 illustrates the proposed mechanism by which green-synthesized silver nanoparticles (AgNPs) mitigate the hazards associated with chlorpyrifos pesticide (S18) [4–8].

The unique properties of AgNPs, including their small size, large surface area, and high reactivity, enable them to interact with pesticide molecules in several ways. As depicted, AgNPs can catalytically degrade the pesticide, breaking down its molecular structure. Additionally, pesticide molecules can adsorb onto the AgNP surface or form complexes with the nanoparticles. These interactions result in a nanocomposite (AgNPs/S18) with beneficial outcomes, such as reduced pesticide concentration, decreased environmental persistence, and lower bioavailability.

This mechanism effectively addresses the potentially harmful effects of chlorpyrifos, including cellular damage, neurotoxicity, and DNA damage [10–12]. By leveraging these nanoscale interactions, the AgNPs/S18 nanocomposite presents a promising approach to mitigating pesticide hazards while maintaining pest control efficacy [30–34].

### Biological activity of AgNPs and nanocomposite S18

#### Colony forming unit counting test (CFU)

We evaluated the antibacterial efficacy of AgNPs and AgNPs/S18 nanocomposite treatments against *Staphylococcus aureus* by quantifying colony-forming units (CFU) under controlled conditions [72, 112, 113]. The data in Table 4 demonstrates significant antibacterial activity for both materials compared to the control: Colony Forming Units (CFU): Both treatments substantially reduced CFU counts. AgNPs showed the highest efficacy, reducing CFU to 10, while AgNP\S18 nanocomposite reduced it to 23, compared to 82 in the control. Total CFU/ml: AgNPs treatment resulted in 500,000 CFU/ml, while AgNPs\S18 nanocomposite yielded 1,150,000 CFU/ml, markedly lower than the control’s 4,100,000 CFU/ml. Log reduction: The log total CFU values (5.70 for AgNPs and 6.06 for AgNPs\S18 nanocomposite) indicate substantial bacterial load reduction compared to the control (6.61). Percentage reduction: AgNPs exhibited superior antibacterial activity with an 87.8% reduction in bacterial population, while AgNPs\S18 nanocomposite achieved a 72% reduction. These results suggest that both materials, particularly AgNPs, show promise as potent antibacterial agents against S. aureus due to their potentially smaller particle size or different surface properties compared to the nanocomposite.

**Fig. 10:**
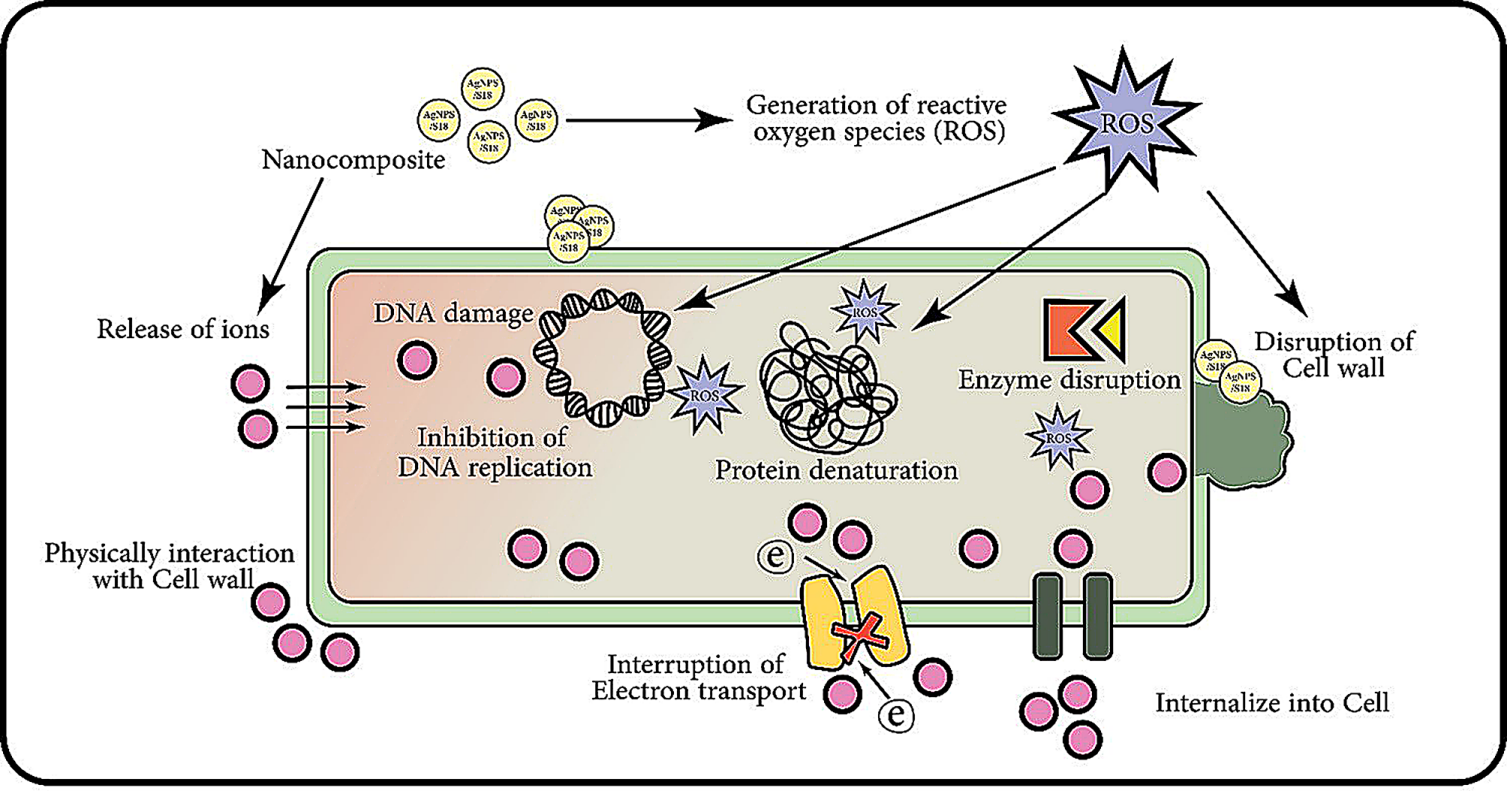
Multifaceted antibacterial mechanisms of AgNPs/S18 nanocomposite

As illustrated in Fig. 10, the nanocomposite initiates a cascade of destructive events upon interaction with bacterial cells. Primarily, it generates reactive oxygen species (ROS), which act as a potent oxidative stress inducer. These ROS and ions released from the nanocomposite penetrate the bacterial cell wall, causing widespread damage. The nanoparticles physically interact with the cell wall, potentially compromising its integrity and facilitating further penetration of antibacterial agents.

Once inside the cell, the nanocomposite’s components wreak havoc on various cellular structures and processes. DNA becomes a primary target, with ROS and released ions causing significant damage to the genetic material. This damage leads to mutations and inhibits DNA replication, halting bacterial reproduction. Simultaneously, the nanocomposite disrupts protein function through denaturation, severely impacting cellular metabolism and structural integrity. Enzyme activity is particularly affected, further crippling the bacteria’s ability to maintain normal functions.

The antibacterial action extends to the cell’s energy production systems as well. The nanocomposite interrupts electron transport chains in the bacterial membrane, compromising the cell’s ability to generate energy. Some nanoparticles may even be internalized completely, allowing for direct interaction with and disrupting intracellular components. The cumulative effect of these mechanisms is the comprehensive breakdown of bacterial cellular functions, leading to cell death.

This multi-pronged approach employed by the AgNPs/S18 nanocomposite makes it a formidable antibacterial agent. The synergistic effect of silver nanoparticles combined with the S18 component enhances overall efficacy, simultaneously targeting bacteria through various pathways. This comprehensive assault on bacterial cells ensures high effectiveness and potentially reduces the likelihood of bacteria developing resistance, as they would need to evolve defences against multiple mechanisms of action concurrently. The diverse and potent antibacterial properties of the AgNPs/S18 nanocomposite thus present a promising solution for combating bacterial infections and contamination across various applications.

**Table 4:** Presents a comparative investigation of the antibacterial effectiveness of AgNPs\S18 nanocomposite and AgNPs against Staphylococcus aureus

Staphylococcus aureus	AgNPs\S18 nanocomposite	AgNPs	Control
Dilution Factor	10^− 4^	10^− 4^	10^− 4^
Volume of broth plated (µl)	20 µl	20 µl	20 µl
Colony Forming Unit (CFU) at the dilution factor	23	10	82
Total CFU /ml	1,150,000	500,000	4,100,000
Log total CFU	6.06	5.70	6.61
%	72%	87.8%	---

The antibacterial activity of silver nanoparticles (AgNPs) synthesized using various plant extracts was evaluated in several separate studies (Table 5) [114, 115]. Raya et al. used Gossypium hirsutum extract to produce AgNPs, demonstrating effectiveness against Xanthomonas campestris and X. axonopodis, although specific inhibition zone measurements were not provided. Lava et al. synthesized AgNPs using Justica wynaadensis leaf extract, showing significant antibacterial activity against both gram-positive and gram-negative bacteria [114]. Against Escherichia coli, these AgNPs produced an inhibition zone of 14 mm at 600 µg/mL concentration, while for Micrococcus luteus, a gram-positive bacterium, the inhibition zone reached 15 mm at 800 µg/mL. our study utilized Psidium guajava leaf extract to create AgNPs and further developed an AgNP/S18 nanocomposite. Our study focused on Staphylococcus aureus and employed a different methodology, measuring antibacterial activity through colony-forming unit (CFU) reduction. The AgNPs showed superior antibacterial efficacy with an 87.8% reduction in bacterial population, while the AgNP/S18 nanocomposite achieved a 72% reduction. These results collectively highlight the broad-spectrum antibacterial efficacy of plant-mediated AgNPs against various bacterial species, demonstrating their potential as powerful antibacterial agents.

**Table 5:** Comparative analysis of antibacterial activity of plant-mediated silver nanoparticles across different studies

Study	AgNP source	Target bacteria	Concentration/method	Antibacterial activity
Ray et al.	Gossypium hirsutum	Xanthomonas campestris	Not specified	Effective (no measurement)
Ray et al.	Gossypium hirsutum	X. axonopodis	Not specified	Effective (no measurement)
Lava et al.	Justica wynaadensis	Escherichia coli	600 µg/mL	14 mm inhibition zone
Lava et al.	Justica wynaadensis	Micrococcus luteus	800 µg/mL	15 mm inhibition zone
Current study	Psidium guajava	Staphylococcus aureus	CFU reduction method	87.8% population reduction (AgNPs)
Current study	Psidium guajava (AgNP/S18 nanocomposite)	Staphylococcus aureus	CFU reduction method	72% population reduction

#### Cytotoxicity evaluation of chlorpyrifos (S18), AgNPs, Nanocomposite (AgNPs\S18) MTT assay method

**Fig. 11:**
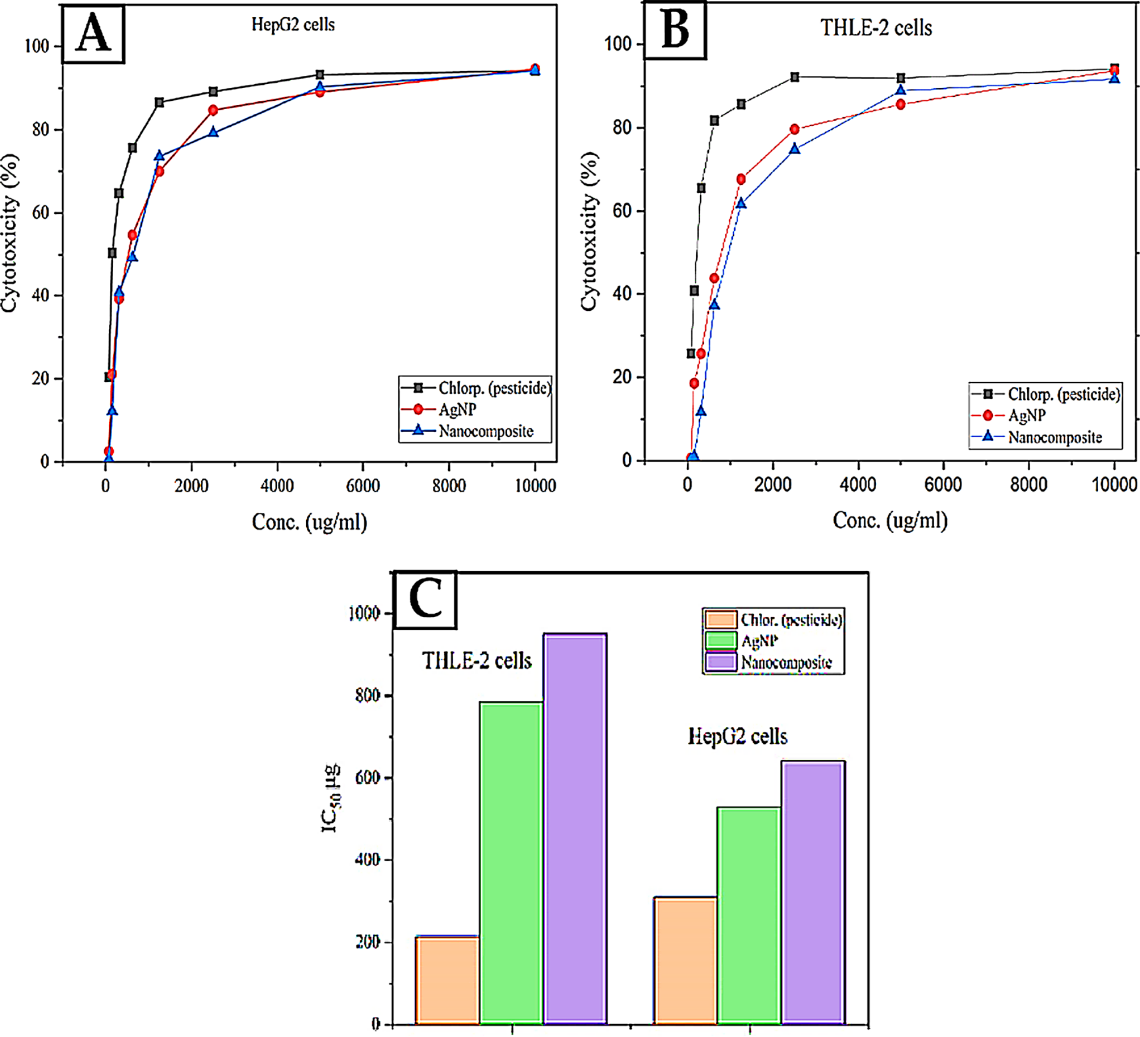
**A** Cytotoxic activity of the AgNPs, S18, and Nanocomposite (AgNPs\S18) against hepatic cellular carcinoma cells (HepG-2). **B** Cytotoxic activity of the AgNPs, S18, and Nanocomposite (AgNPs\S18) against a regular human liver epithelial cell line (THLE-2). **C** (IC_50_) of the green synthesized AgNPs, S18, and Nanocomposite (AgNPs\S18) against (HepG-2) and (THLE-2)

The MTT assay evaluated the cytotoxicity observed in vitro of various compounds on two cell lines, THLE2 (human liver epithelial cells) and HepG2 (human hepatocellular carcinoma cells) [74, 116, 117]. The compounds investigated included (S18), (AgNPs), and a Nanocomposite of (AgNPs and S18). The pesticide chlorpyrifos exhibited concentration-dependent cytotoxicity on both cell lines. In THLE2 cells, chlorpyrifos displayed an IC_50_ value of 214.31 µg/mL, indicating that 50% of the cells were viable at this concentration (Fig. 11B). Similarly, in HepG2 cells, the IC_50_ value for chlorpyrifos was 310.07 µg/mL, as shown in (Fig. 11A). These results suggest that chlorpyrifos exerts toxic effects on liver cells, with HepG2 cancer cells being slightly more resistant than normal THLE2 cells. AgNPs alone demonstrated potent cytotoxicity, with IC_50_ values of 785.36 µg/mL and 529.74 µg/mL for THLE2 and HepG2 cells, respectively. Notably, the combination of AgNPs and S18 exhibited enhanced cytotoxicity compared to AgNPs alone, with lower IC_50_ values of 838.66 µg/mL and 711.71 µg/mL for THLE2 and HepG2 cells, respectively, as shown in (Fig. 11C). This synergistic effect suggests that the combination of AgNPs and S18 may have potential applications in targeted cancer therapy or as antimicrobial agents, where their combined cytotoxicity could selectively kill cancer cells or microbial pathogens while minimizing harm to normal cells. Interestingly, the data also revealed that HepG2 cancer cells exhibit more susceptibility to the cytotoxic effects of AgNPs and the AgNPs-S18 combination than normal THLE2 cells. This observation could be attributed to the inherent differences in cellular metabolism, proliferation rates, and the potential mechanisms of action of the tested compounds on cancer cells versus normal cells. Overall, these findings offer valuable visions into the cytotoxic profiles of the compounds tested and their potential applications in various fields of study, including toxicology, nanomedicine, environmental studies, and cancer research [118]. Detailed information on the concentrations used in biological applications, including IC_50_ values for Silver Nanoparticles (AgNPs) and AgNPs/S18 Nanocomposite on THLE2 and HepG2 cell lines, as well as calculations for estimated safe concentrations, is provided in the Supplementary Data section under ‘Concentrations for Biological Applications’.

While our study focused primarily on the synthesis and characterization of AgNPs using Psidium guajava leaf extract, it is essential to consider the potential anticancer applications of these nanoparticles. Recent studies have shown promising results in this area.

Lava et al. (2021) reported significant anticancer activity of AgNPs synthesized using Justica wynaadensis leaf extract against A549 lung cancer cells [114]. Their study showed an IC_50_ value of 60 µg/mL, indicating potent cytotoxicity against these cancer cells. Ahn and Park (2020) demonstrated the significant anticancer potential of AgNPs green-synthesized using plant extracts against A549 lung cancer and HeLa cervical cancer cells [119]. Their study showed that AgNPs synthesized using extracts from Ardisia incarnata, Maesa calophylla, and Maesa laxiflora exhibited particularly high cytotoxicity, with cell viabilities as low as 10.9%, 8.1%, and 25.6% respectively for A549 cells at 100 µM concentration. These AgNPs also induced substantial reactive oxygen species (ROS) generation, up to an 11.6-fold increase compared to control, and caused cell cycle arrest predominantly in the S phase. Table 6 compares the anticancer activities of AgNPs synthesized using various plant extracts.

**Table 6:** Comparison of anticancer activities of plant-mediated synthesized AgNPs

Plant extract	Sample	Normal/Cancer Cell Line	IC50 (µg/mL)	Reference
Justica wynaadensis	AgNPs	A549 (Lung)	60	Lava et al. [114]
Psidium guajava	AgNPs	THLE2	785.36	Current study
Psidium guajava	AgNPs	HepG2	529.74	Current study
-	S18	THLE2	214.31	Current study
-	S18	HepG2	310.07	Current study
Psidium guajava	Nanocomposite (AgNPs\S18)	THLE2	838.66	Current study
Psidium guajava	Nanocomposite (AgNPs\S18)	HepG2	711.71	Current study
Ardisia incarnata	AgNPs-29	A549 (Lung)	~ 13.6*	Ahn and Park [119]
Ardisia incarnata	AgNPs-29	HeLa (Cervical)	~ 47.8*	Ahn and Park [119]
Maesa calophylla	AgNPs-33	A549 (Lung)	~ 10.1*	Ahn and Park [119]
Maesa calophylla	AgNPs-33	HeLa (Cervical)	~ 57.9*	Ahn and Park [119]
Maesa laxiflora	AgNPs-47	A549 (Lung)	~ 32.0*	Ahn and Park [119]
Maesa laxiflora	AgNPs-47	HeLa (Cervical)	~ 82.5*	Ahn and Park [119]

*The IC_50_ values for the Ahn and Park [119] study are rough estimates based on the cell viability percentages at 100 µM concentration

**Fig. 12:**
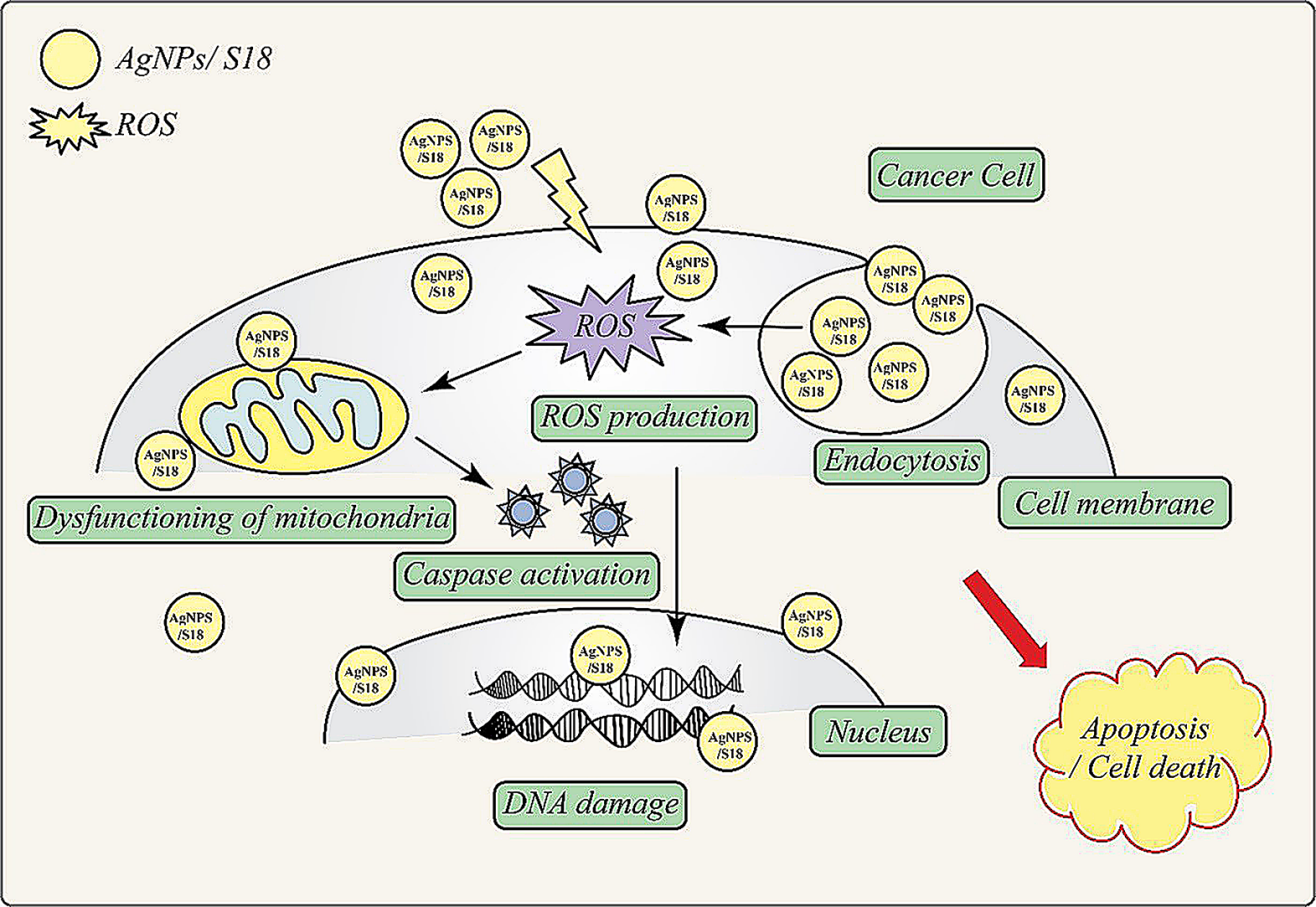
Anticancer mechanisms of AgNPs/S18 nanocomposite

The AgNPs/S18 nanocomposite exhibits a powerful anticancer effect through multiple simultaneous mechanisms, as illustrated in Fig. 12. Upon interacting with cancer cells, it generates reactive oxygen species (ROS), which induce significant oxidative stress. The nanocomposite also releases ions that, along with ROS, penetrate the cell membrane to cause widespread intracellular damage [114].

Inside the cell, the nanocomposite targets various critical components. It causes DNA damage, inhibits replication and halts cancer cell proliferation. Protein denaturation and enzyme disruption severely impact cellular metabolism and structural integrity. The nanocomposite also interrupts mitochondrial electron transport, compromising energy production and potentially triggering apoptosis.

Physical interaction with the cell membrane increases its permeability, facilitating further entry of anticancer agents. This multifaceted approach leads to the activation of various cell death pathways, including apoptosis and necrosis.

The synergistic effect of silver nanoparticles and the S18 component enhances overall efficacy against cancer cells. This comprehensive assault ensures high effectiveness and potentially reduces the likelihood of cancer cells developing resistance, as they would need to evolve defences against multiple mechanisms simultaneously.

Moreover, the nanocomposite’s ability to generate ROS may be particularly effective against cancer cells, which often have higher baseline ROS levels and may be more susceptible to additional oxidative damage than normal cells. This potential for selective toxicity makes the AgNPs/S18 nanocomposite a promising candidate for cancer therapy, offering a powerful approach to overcoming challenges such as drug resistance while potentially sparing normal cells.

### Effect of S18, AgNPs, and nanocomposite (AgNPs\S18) on Sprague-Dawley rats

#### Effect of S18, AgNPs, and nanocomposite (AgNPs\S18) on the liver and kidney

**Fig. 13:**
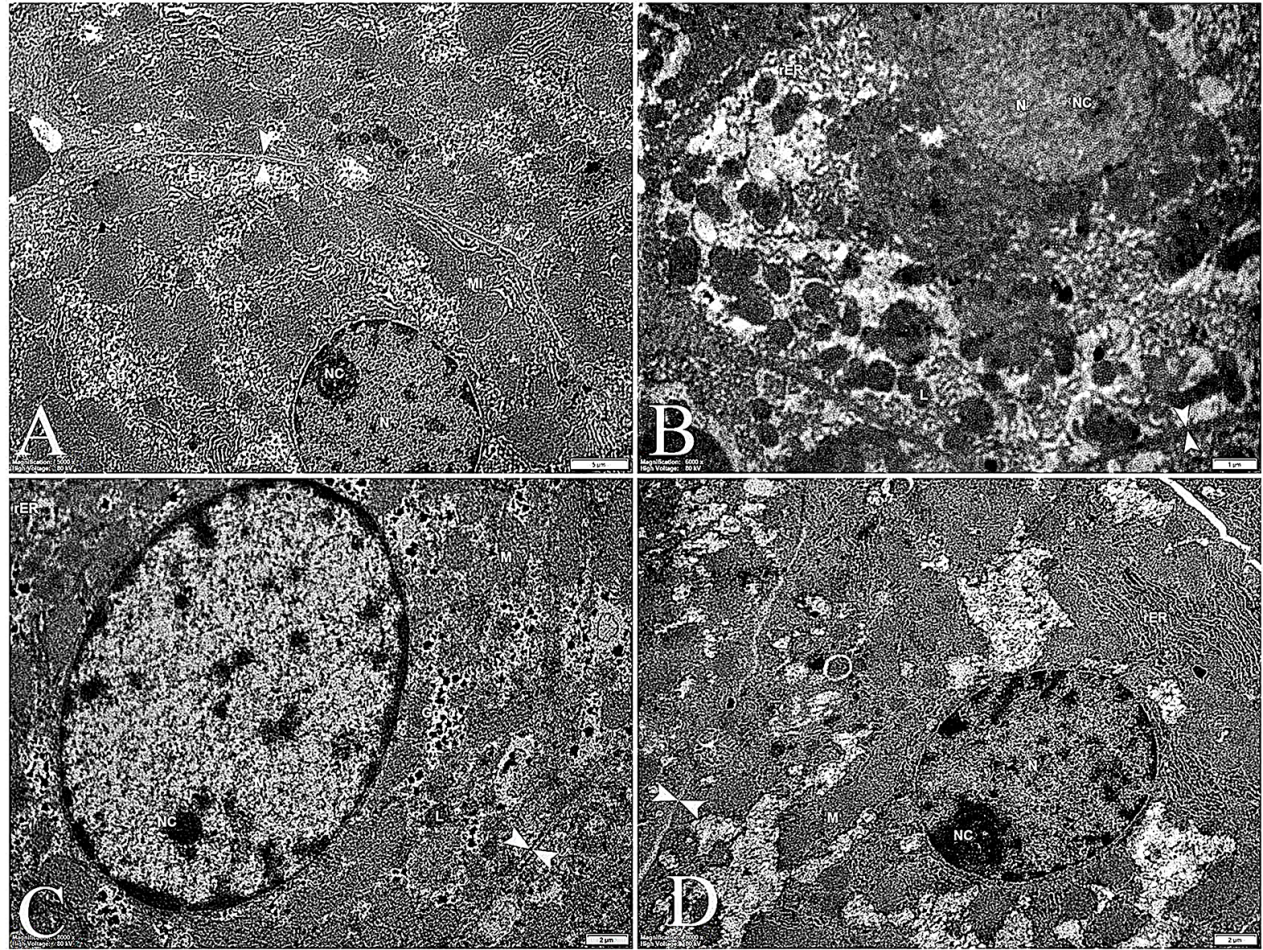
Transmission Electron Microscope of the liver. **A** Electron microscopic picture of the control liver showing a hepatocyte with large vesicular nucleus (N) and prominent nucleolus (NC). The clear cell boundary (between arrowheads) surrounds the cytoplasm rich in mitochondria (MI), glycogen granules (Gg), and rough endoplasmic reticulum (rER). **B** The organic phosphate group shows pyknosis of the nuclei and less differentiated nucleoli, hydropic vacuoles (V), lysosomes (L), and dilation of the endoplasmic reticulum. **C** The nano-treated group was quite like the control group. The nucleus and nucleolus are apparent. The cell boundaries are clear, with the cytoplasm containing minimal vacuolations and lysosomes. The rough endoplasmic reticulum and cytoplasmic glycogen granules are normal. **D** The composite liver is close to the nano group

Figure 13 A shows transmission electron microscopy of the control liver. The hepatocytes in the micrograph have polygonal shapes, and their nuclei are located centrally. The cytoplasmic matrix is apparent, and there are many mitochondria present. The sections also show a solid granular endoplasmic reticulum [120–122]. The diseased group (Fig. 13B) shows vigorous ultrastructural changes, including pyknotic nuclei, hydropic vacuoles in the perinuclear area, lysosomes, and dilation of the endoplasmic reticulum [123]. The nano-treated group (Fig. 13C) was similar to the control group.

The nucleus and nucleolus are apparent. The cell boundaries are clear, with the cytoplasm containing minimal vacuolations and lysosomes. The rough endoplasmic reticulum and cytoplasmic glycogen granules are more or less normal.

Liver sections of the composite group (Fig. 13D) were nearly regular in ultrastructural architecture.

**Fig. 14:**
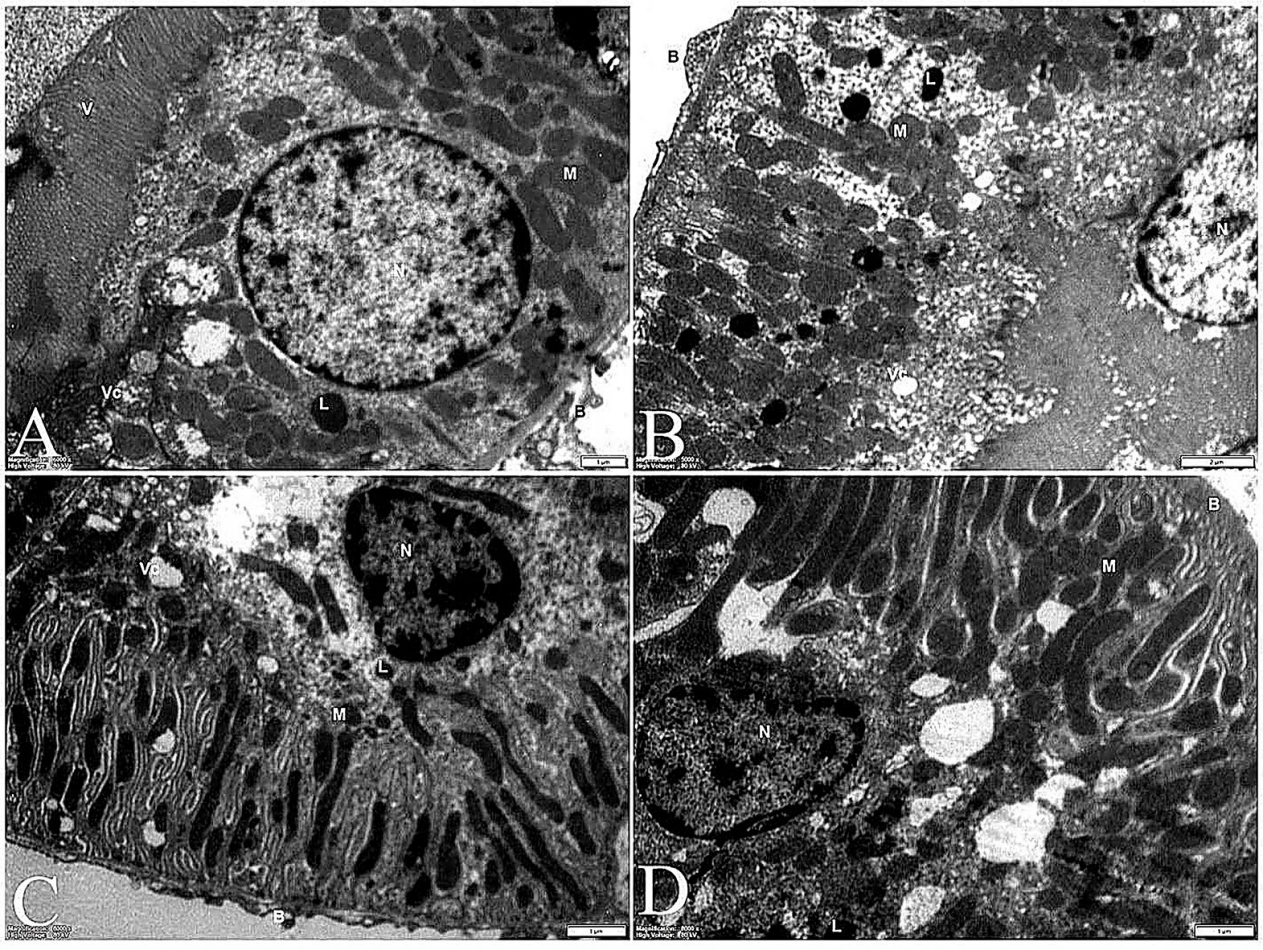
TEM pictures of the kidney. **A** These TEM pictures of the kidney’s proximal tubules represent typical endothelial cell lining. The cell has a rounded nucleus (N), an abundant Bruch border (B), basal infoldings (B), extensive mitochondria (M), lysosomes (L), and endocytic vesicles (Vc). **B** The diseased group shows distorted nuclei, irregularly distributed mitochondria, more frequent lysosomes, and irregular basal infoldings. **C** The nano-treated group shows recovery of the mitochondria, which appear more frequent. Lysosomes and endocytic vesicles are less numerous. **D** The composite group is close to the nano group

TEM Analysis of Kidney Proximal Tubules under different conditions provides insights into cellular structures and their changes in response to disease and treatment [124]. Control Group (Normal Kidney Tissue) (Fig. 14A) The TEM image of healthy proximal tubules reveals the typical ultrastructure of endothelial cells, Nucleus (N) A prominent, rounded structure containing the genetic material, Brush border (B) An extensive array of microvilli on the apical surface, increasing the cell’s surface area for absorption, Basal infoldings: Complex membrane invaginations at the cell’s base, associated with ion transport [125].Mitochondria (M): Numerous elongated organelles indicating high metabolic activity [126]. Lysosomes (L): Membrane-bound organelles containing digestive enzymes. Endocytic vesicles (Vc): Small, membrane-enclosed compartments involved in cellular uptake of molecules.

Diseased Group (Fig. 14B) The TEM image of diseased proximal tubules shows significant alterations in cellular structure: Distorted nuclei and irregular shape, possibly indicating cellular stress or damage [127, 128]. Irregularly distributed mitochondria: Abnormal arrangement, suggesting disrupted energy production. Increased frequency of lysosomes may indicate enhanced cellular breakdown or attempted repair processes. Irregular basal infoldings: Disruption in the regular folding pattern, potentially affecting ion transport. Nano-treated Group (Fig. 14C) This group shows signs of recovery following treatment with nanoparticles: Recovery of mitochondria: Increased number and more normal distribution, suggesting improved cellular energy production. Reduced lysosomes and endocytic vesicles: Indicate a decrease in cellular stress and return to more normal function. Overall, the cellular structure appears more organized than the diseased group’s. Composite Group (Fig. 14D**)**, likely treated with a combination of nanoparticles and other agents, shows similarities to the nano-treated group: Cellular structures resemble those in the nano-treated group with potential synergistic effects of combined treatments, resulting in improved cellular recovery.

#### Histopathological effect of S18, AgNPs, and nanocomposite (AgNPs\S18) on the liver and kidney tissues

**Fig. 15:**
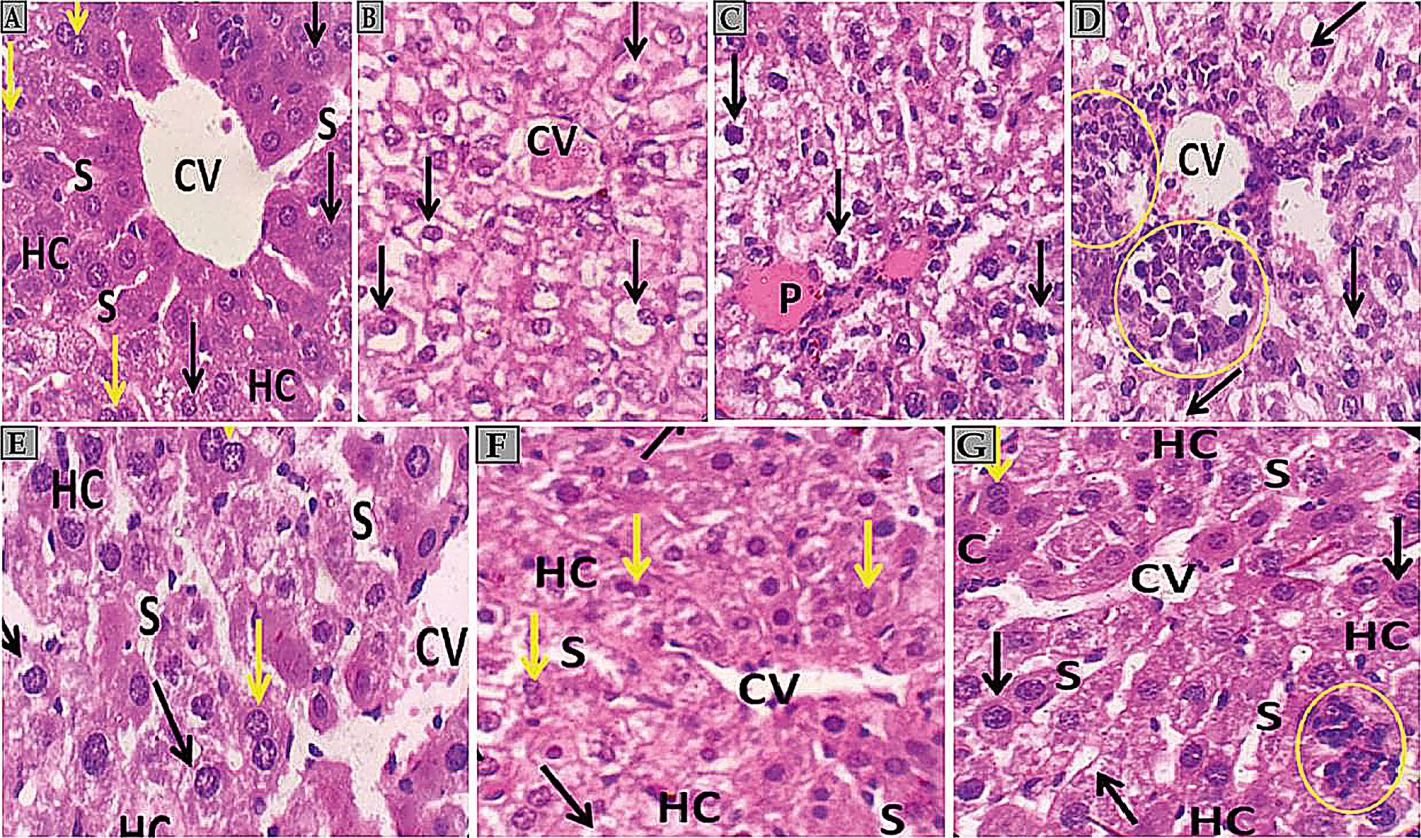
**A** Photomicrographs of H and E-stained liver sections from the control group. Liver sections from the control group show anastomosing hepatic cords (HC) radiating from the central vein (CV) and separated by blood sinusoids (S). The polygonal hepatocytes have eosinophilic cytoplasm and central rounded vesicular nuclei. (black arrows). Some hepatocytes are binucleated (yellow arrows). **B** liver sections from the **diseased group** show congested central veins (CV) and disorganization of the hepatic cords. Widespread vacuolated hepatocytes (arrows) with shrunken, irregular, and darkly stained pyknotic nuclei are noticed. **C** liver sections from the **diseased group** show congested hepatic portal veins (P) and widespread vacuolated hepatocytes (arrows) with shrunken, irregular, and darkly stained pyknotic nuclei. **D** liver sections from the **diseased group** show disorganization of the hepatic cords and widespread vacuolated hepatocytes (arrows) with shrunken, irregular, and darkly stained pyknotic nuclei. Severe mononuclear inflammatory cell infiltration (circles) appears close to the central vein (CV). **E** The liver section from the treated group shows regular anastomosing hepatic cords (HC) radiating from the central vein (CV) and separated by blood sinusoids (S). Most hepatocytes have eosinophilic cytoplasm and central rounded vesicular nuclei (yellow arrows). Some vacuolated hepatocytes (black arrows) with shrunken, irregular pyknotic nuclei are also detected. **F** The liver section from the treated group shows regular anastomosing of hepatic cords (HC) radiating from the central vein (CV) and separated by blood sinusoids (S). Most hepatocytes appear normal with eosinophilic cytoplasm and central rounded vesicular nuclei (yellow arrows). A few vacuolated hepatocytes (black arrows) with shrunken and irregular pyknotic nuclei were also detected. **G** the liver section from the best-treated group shows regular anastomosing hepatic cords (HC) radiating from the central vein (CV) and separated by blood sinusoids (S). Hepatocytes appear normal with eosinophilic cytoplasm and central rounded vesicular nuclei (yellow arrows). A few inflammatory cell infiltrations between hepatic cords are also noted

Photomicrographs of H and E-stained liver sections reveal distinct characteristics across control, diseased, and treated groups [129, 130]. The control group shows standard liver architecture with anastomosing hepatic cords radiating from the central vein, separated by blood sinusoids [131]. Hepatocytes appear polygonal with eosinophilic cytoplasm and central rounded vesicular nuclei, some binucleated (Fig. 15A). In contrast, the diseased group exhibits congested central and portal veins, disorganized hepatic cords, and widespread vacuolated hepatocytes with shrunken, irregular, and darkly stained pyknotic nuclei [132]. Severe mononuclear inflammatory cell infiltration is observed near the central vein (Fig. 15B, C, and D). The treated group shows improvement, with most hepatocytes appearing regular and hepatic cords regaining their structure, though some vacuolated hepatocytes with pyknotic nuclei are still present (Fig. 15E and F). The best-treated group demonstrates near-normal liver histology, with only a few inflammatory cell infiltrations between hepatic cords (Fig. 15G). All observations were made at 400x magnification using H and E staining.

**Fig. 16:**
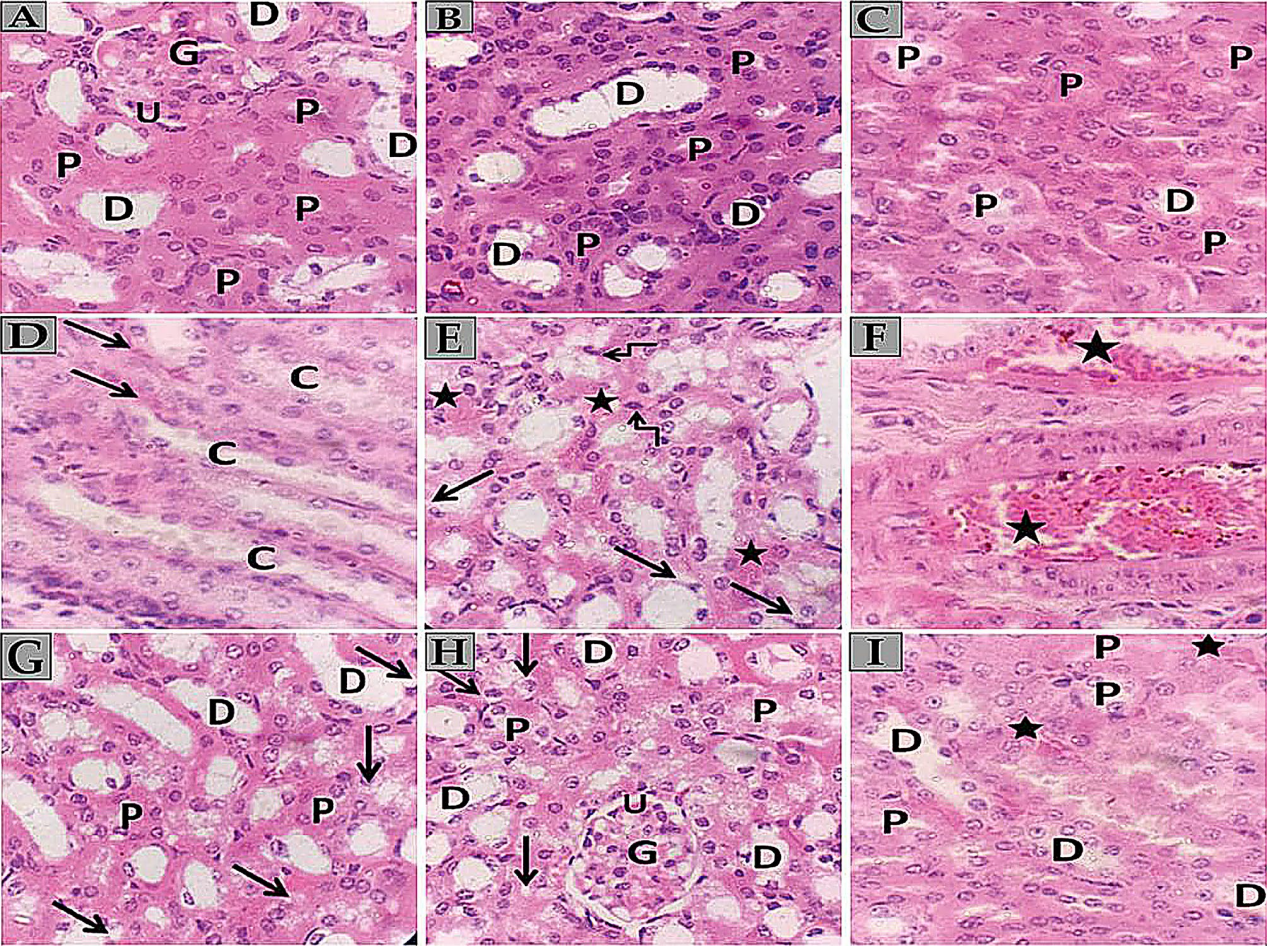
**A** The kidney section from the control group shows a renal cortex with normal glomerulus (G) and regular capsular space (U). Narrow proximal convoluted tubules (P) and wider distal convoluted tubules (D) are lined by simple cuboidal epithelium with eosinophilic cytoplasm and central rounded vesicular nuclei. **B** and **C** Kidney sections from the control group display normal renal cortex with narrow proximal convoluted tubules (P) and wider distal convoluted tubules (D) lined by simple cuboidal epithelium with eosinophilic cytoplasm and central rounded vesicular nuclei. **D** The control group kidney section shows a normal renal medulla containing collecting tubules (C) lined by simple cubical epithelium and separated by minimal interstitium (arrows) with peritubular capillaries. **E** Diseased group kidney section reveals vacuolar degeneration (arrows) in the cytoplasm of convoluted tubules, congestion of peritubular capillaries (stars) within the interstitial space, and some tubular cells appearing shrunken with deeply stained eosinophilic cytoplasm and pyknotic nuclei (angled arrows) [H and E, x400]. **F** The diseased group kidney section shows congestion and dilatation of peritubular capillaries (stars) within the interstitial space. **G** The treated group kidney section demonstrates potentially alleviated renal histoarchitecture with normal proximal (P) and distal (D) convoluted tubules and some vacuolated cells lining the renal tubules (arrows). **H** The treated group kidney section shows potentially alleviated renal histoarchitecture with normal renal glomeruli (G), urinary space (U), normal proximal (P) and distal (D) convoluted tubules, some vacuolated cells lining the renal tubules (arrows), and few shrunken tubular cells with deeply stained pyknotic nuclei (angled arrows). **I** Treated group kidney section reveals potentially alleviated renal histoarchitecture containing normal proximal (P) and distal (D) convoluted tubules separated by minimal interstitium with slightly congested peritubular capillaries (stars)

Photomicrographs of H and E-stained kidney sections (Fig. 16) reveal distinct features across control, diseased, and treated groups. The control group demonstrates standard renal architecture, with the cortex showing typical glomeruli, regular capsular spaces, and well-defined proximal and distal convoluted tubules lined by simple cuboidal epithelium. The renal medulla contains collecting tubules with minimal interstitium and peritubular capillaries. In contrast, the diseased group exhibits significant alterations, including vacuolar degeneration in convoluted tubules, congestion and dilation of peritubular capillaries, and some tubular cells appearing shrunken with pyknotic nuclei.

The treated group demonstrates potential alleviation of renal histoarchitecture, with primarily normal glomeruli and convoluted tubules. However, some vacuolated cells and pyknotic nuclei are still observed in tubular linings, along with slightly congested peritubular capillaries. These observations, made at 400x magnification using H and E staining, suggest a partial recovery in the treated group compared to the diseased state, though some residual damage remains evident.

#### Liver and kidney functions

**Fig. 17:**
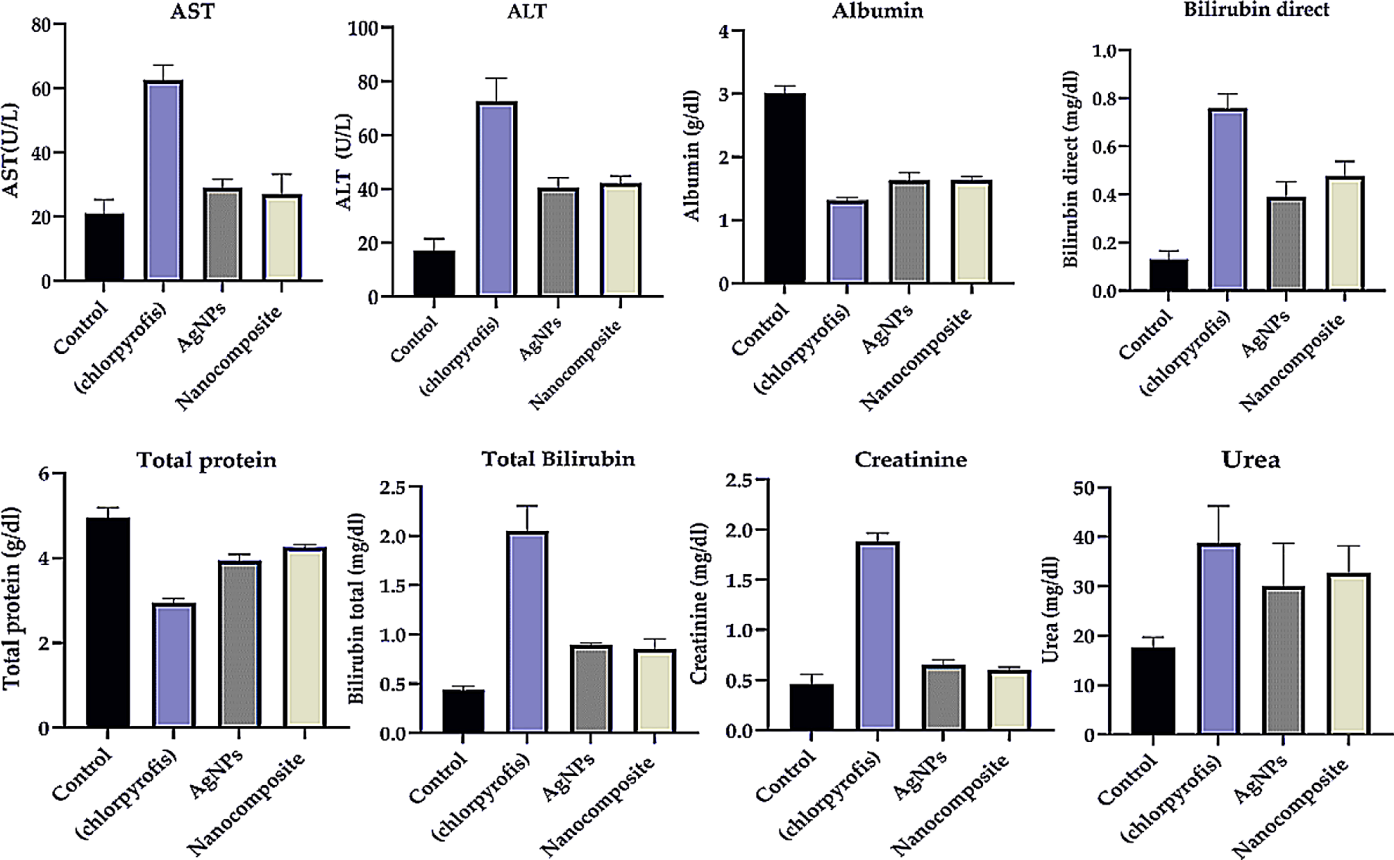
Liver and kidney functions

The Sprague Dawley albino rats’ liver functions were tested by measuring ALT, AST, Total protein, and Albumin. Kidney functions were also tested by measuring Creatinine and urea (Fig. 17). The study compared liver function indicators across control, organophosphate-exposed, and nanoparticle-treated groups. Control samples showed normal liver function, with ALT, AST, protein, albumin, and bilirubin levels within healthy ranges. Organophosphate exposure significantly impaired liver function, evidenced by markedly elevated ALT and AST levels, severely reduced protein synthesis, and disrupted bilirubin metabolism. Nanosilver and silver nanocomposite treatments also affected liver function to a lesser extent than organophosphate [133]. These nanoparticles caused moderate increases in ALT and AST, less pronounced reductions in protein synthesis, and only slight elevations in bilirubin levels [134]. The results suggest that while nanoparticle treatments have some hepatotoxic effects, they protect against the severe liver dysfunction caused by organophosphate exposure. The study compared kidney function indicators. The control group exhibited normal creatinine and urea levels, which indicated healthy kidney function. In stark contrast, organophosphate treatment led to significantly elevated creatinine and urea levels, suggesting severe renal impairment or reduced glomerular filtration rate. The nanosilver and silver nanocomposite treatments resulted in moderate increases in these renal markers, indicating some degree of kidney stress but considerably less than that caused by organophosphate exposure. These findings suggest that while nanoparticle treatments may have some renal effects, they appear less nephrotoxic than organophosphates and could protect against severe kidney dysfunction.

### Comparison with other green synthesis methods

To contextualize our findings, we compared our green synthesis method using Psidium guajava leaf extract with other recent studies using various plant extracts for nanoparticle synthesis (Table 7). Our method produced silver nanoparticles (AgNPs) with an average size of 9.038 nm (XRD) or 7.531 nm (DLS) using Psidium guajava (guava) leaf extract. These AgNPs demonstrated antibacterial activity against Staphylococcus aureus and showed cytotoxicity against THLE2 and HepG2 cell lines. The small size of our AgNPs is particularly noteworthy, as it may contribute to their enhanced biological activities. In comparison, Basavegowda et al. [20] synthesized gold nanoparticles (AuNPs) using Artocarpus heterophyllus (jackfruit) fruit extract [20]. Their AuNPs had an average size of 20–25 nm, more significant than our AgNPs. These AuNPs exhibited a mixture of spherical and polyhedron shapes and showed antibacterial activity against E. coli and Streptobacillus. Using a different plant extract and metal precursor resulted in distinct nanoparticle characteristics and biological activities. Basavegowda et al. [22] used Saururus chinensis leaf extract to synthesize palladium nanoparticles (PdNPs) [22]. These PdNPs were significantly smaller than our AgNPs and the AuNPs from the jackfruit study, with a mean size of approximately 4 nm. The PdNPs were spherical and exhibited moderate antioxidant activity (DPPH: IC_50_ 26.66 µg/mL) and potent anti-tyrosinase activity (85.95% at 100 µg/mL).

**Table 7:** Comparison of green synthesis methods for metal nanoparticles using various plant extracts

Study	Plant extract	Type of nanoparticle	Particle size (nm)	Shape	Key activities/Properties
Current work	Psidium guajava (Guava)	AgNPs	9.038 (XRD), 7.531 (DLS)	Spherical	Antibacterial against S. aureus; Cytotoxicity against THLE2 and HepG2 cells
Sana et al. [21]	Perilla frutescens crude polysaccharides (PFCPS)	AgNPs	18.7–23.8	Spherical	Antioxidant (DPPH, ABTS, Reducing power); Antibacterial against E. coli and K. pneumoniae
Basavegowda et al. [20]	Artocarpus heterophyllus Lam. (Jackfruit) fruit extract	AuNPs	20–25 (small spherical), 5 μm (large polyhedron)	A mixture of spherical and polyhedron	Antibacterial against E. coli and Streptobacillus; Synergistic effect with antibiotics
Basavegowda et al. [22]	Saururus chinensis leaf extract	PdNPs	~ 4	Spherical	Antioxidant (DPPH: IC_50_ 26.66 µg/mL); Anti-tyrosinase (85.95% at 100 µg/mL)

This study demonstrates how different plant extracts can form nanoparticles with varying sizes and biological properties. Our study stands out for its focus on cytotoxicity evaluations, which were not reported in the other two studies. This additional data provides valuable insights into the potential biomedical applications and safety profile of our synthesized AgNPs. Furthermore, our use of guava leaf extract for AgNP synthesis represents a novel approach compared to the jackfruit and S. chinensis extracts used in the other studies. In conclusion, these comparisons highlight the versatility of green synthesis methods and the significant impact the choice of plant extract and metal precursor can have on the resulting nanoparticles’ characteristics and biological activities. Our process offers potential advantages in particle size and provides valuable cytotoxicity data, contributing to the advancement of eco-friendly nanoparticle synthesis techniques for biomedical applications.

## Conclusion

The green synthesis of AgNPs using Psidium guajava leaf extract offers an eco-friendly and efficient technique for producing AgNPs and AgNP/S18 nanocomposites with significant antibacterial and cytotoxic properties. This study demonstrates the potential of these nanoparticles to reduce the harmful effects of organophosphorus pesticides, particularly in mitigating toxicity in liver and kidney tissues. Our comprehensive characterization confirmed the successful synthesis of AgNPs and the formation of a stable Nanocomposite (AgNPs/S18). Antibacterial assays revealed significant activity against Staphylococcus aureus, with AgNPs and the Nanocomposite (AgNPs/S18) showing 87.8% and 72% reduction in bacterial population, respectively. Cytotoxicity studies indicated enhanced Nanocomposite (AgNPs/S18) efficacy compared to AgNPs alone in normal liver and liver cancer cell lines, suggesting potential applications in targeted therapies. In vivo studies on Sprague-Dawley rats demonstrated both AgNPs and the Nanocomposite (AgNPs/S18) protective effects against Chlorpyrifos-induced toxicity in liver and kidney tissues. Histopathological and ultrastructural analyses provided evidence of reduced cellular damage in treated rats, particularly with the Nanocomposite (AgNPs/S18). These results highlight the potential of integrating nanotechnology with traditional pesticide management strategies to develop safer and more sustainable agricultural practices. The Nanocomposite (AgNPs/S18) shows promise in reducing environmental and health risks associated with conventional organophosphorus pesticides while maintaining efficacy in pest control. However, further research is necessary to fully comprehend the long-term effects of these nanoparticles on various ecosystems and to optimize their application in real-world agricultural settings. In conclusion, this study contributes significantly to sustainable agriculture and environmental protection. Future research should focus on optimizing large-scale production, investigating long-term environmental impact, exploring similar nanocomposites with other pesticides, conducting field trials, and elucidating the mechanisms of pesticide toxicity mitigation. This work represents a crucial step towards developing more environmentally friendly and health-conscious approaches to pest management, aligning with global efforts to promote sustainable agriculture and reduce the negative impacts of conventional pesticides on human health and ecosystems.

## Electronic Supplementary Material

Below is the link to the electronic supplementary material.

Supplementary Material 1

Supplementary Material 2

## Data Availability

The datasets used and/or analysed during the current study are available from the corresponding author on reasonable request.

## Abbreviations

AgNPs: Silver Nanoparticles
CFU: Colony Forming Unit
FTIR: Fourier-Transform Infrared Spectroscopy
MTT: 3-(4,5-Dimethylthiazol-2-yl)-2,5-Diphenyltetrazolium Bromide
SPR: Surface Plasmon Resonance
XRD: X-ray Diffraction
S18: Chlorpyrifos Pesticide
AChE: Acetylcholinesterase
HepG2: Human hepatocellular carcinoma cells
ADHD: Attention deficit hyperactivity disorder
ALT: Alanine transaminase
NC: Prominent nucleolus
(L): Lysosomes
Gg: Glycogen granules
Vc: Endocytic vesicles
(S): Blood sinusoids
(P): Narrow proximal convoluted tubules
AFM: Atomic Force Microscope
DLS: Dynamic Light Scattering
H and E: Hematoxylin and Eosin
PBS: Phosphate-Buffered Saline
TEM: Transmission Electron Microscope
NP: Nanoparticles
ANOVA: Analysis of Variance
N: Vesicular nucleus
Vis: Visible
EPA: Environmental Protection Agency
AST: Aspartate aminotransferase
MI: Mitochondria
(B): Brush border / basal infoldings
rER: Rough endoplasmic reticulum
(CV): The central vein
(D): Wider distal convoluted tubules
(C): Normal renal medulla containing collecting tubules
BET: Brunauer-Emmett-Teller
EDX: Energy-Dispersive X-ray Spectroscopy
IC50: Inhibitory Concentration 50%
SEM: Scanning Electron Microscope
THLE2: Human Liver Epithelial Cells
UV-Vis: Ultraviolet-Visible Spectroscopy
RPM: Revolutions Per Minute
UV: Ultraviolet
FWHM: Full-width at half maximum
DMSO: Dimethyl sulfoxide
PS 80: Polysorbate 80
DA: Dubinin-Astakhov
(G): Normal glomerulus
(V): Hydropic vacuoles
(HC): Hepatic cords
(U): Regular capsular space
(U): Urinary space
