# Supplementary material for: Eco-friendly green synthesis of silver nanoparticles from guajava leaves extract for controlling organophosphorus pesticides hazards, characterization, and in-vivo toxicity assessment
Source: BMC Pharmacol Toxicol. 2024 Dec 18;25:98. doi: 10.1186/s40360-024-00826-7 (PMC11658244; doi:10.1186/s40360-024-00826-7)
Supplement: Supplementary file 1 — Supplementary Material 1 [file 40360_2024_826_MOESM1_ESM.docx]

**List of abbreviations**

| **Abbreviation** | **Full Term** | **Abbreviation** | **Full Term** | **Abbreviation** | **Full Term** |
| --- | --- | --- | --- | --- | --- |
| AgNPs | Silver Nanoparticles | AFM | Atomic Force Microscope | BET | Brunauer-Emmett-Teller |
| CFU | Colony Forming Unit | DLS | Dynamic Light Scattering | EDX | Energy-Dispersive X-ray Spectroscopy |
| FTIR | Fourier-Transform Infrared Spectroscopy | H and E | Hematoxylin and Eosin | IC50 | Inhibitory Concentration 50% |
| MTT | 3-(4,5-Dimethylthiazol-2-yl)-2,5-Diphenyltetrazolium Bromide | PBS | Phosphate-Buffered Saline | SEM | Scanning Electron Microscope |
| SPR | Surface Plasmon Resonance | TEM | Transmission Electron Microscope | THLE2 | Human Liver Epithelial Cells |
| XRD | X-ray Diffraction | NP | Nanoparticles | UV-Vis | Ultraviolet-Visible Spectroscopy |
| S18 | Chlorpyrifos Pesticide | ANOVA | Analysis of Variance | RPM | Revolutions Per Minute |
| AChE | Acetylcholinesterase | N | Vesicular nucleus | UV | Ultraviolet |
| HepG2 | Human hepatocellular carcinoma cells | Vis | Visible | FWHM | Full-width at half maximum |
| ADHD | Attention deficit hyperactivity disorder | EPA | Environmental Protection Agency | DMSO | Dimethyl sulfoxide |
| ALT | Alanine transaminase | AST | Aspartate aminotransferase | PS 80 | Polysorbate 80 |
| NC | Prominent nucleolus | MI | Mitochondria | DA | Dubinin-Astakhov |
| (L) | Lysosomes | (B) | Brush border / basal infoldings | (G) | Normal glomerulus |
| Gg | Glycogen granules | rER | Rough endoplasmic reticulum | (V) | Hydropic vacuoles |
| Vc | Endocytic vesicles | (CV) | The central vein | (HC) | Hepatic cords |
| (S) | Blood sinusoids | (D) | Wider distal convoluted tubules | (U) | Regular capsular space |
| (P) | Narrow proximal convoluted tubules | (C) | Normal renal medulla containing collecting tubules | (U) | Urinary space |
