# Supplementary material for: Eco-friendly green synthesis of silver nanoparticles from guajava leaves extract for controlling organophosphorus pesticides hazards, characterization, and in-vivo toxicity assessment
Source: BMC Pharmacol Toxicol. 2024 Dec 18;25:98. doi: 10.1186/s40360-024-00826-7 (PMC11658244; doi:10.1186/s40360-024-00826-7)
Supplement: Supplementary file 2 — Supplementary Material 2 [file 40360_2024_826_MOESM2_ESM.docx]

**Concentrations Used in Biological Applications**

In our biological assays, we determined the IC_50_ values for both Silver Nanoparticles (AgNPs) and the AgNPs/S18 Nanocomposite on THLE2 (normal liver cells) and HepG2 (liver cancer cells). The IC_50_ values, representing the concentration at which 50% of cells remain viable, were as follows:

Silver Nanoparticles (AgNPs):

- THLE2 cells: 785.36 μg/mL
- HepG2 cells: 529.74 μg/mL

AgNPs/S18 Nanocomposite:

- THLE2 cells: 838.66 μg/mL
- HepG2 cells: 711.71 μg/mL

For safety considerations, we used the lower IC_50_ value between the two cell lines for each compound as the reference point, representing the more sensitive response. Applying a conservative safety factor of 1000, which is commonly used in toxicology to account for interspecies differences and human variability, we calculated the following estimated safe concentrations:

- AgNPs: 529.74 μg/mL ÷ 1000 ≈ 0.53 μg/mL
- AgNPs/S18 Nanocomposite: 711.71 μg/mL ÷ 1000 ≈ 0.71 μg/mL

These calculated values (0.53 μg/mL for AgNPs and 0.71 μg/mL for AgNPs/S18 Nanocomposite) were used to estimate potentially safe concentrations in our biological applications. It's important to note that these are conservative estimates, and actual safe levels may vary depending on the specific application and exposure route.
